# Supplementary material for: Leveraging Synthetic Virology for the Rapid Engineering of Vesicular Stomatitis Virus (VSV)
Source: Viruses. 2024 Oct 21;16(10):1641. doi: 10.3390/v16101641 (PMC11512388; doi:10.3390/v16101641)
Supplement: Supplementary file 1 [file viruses-16-01641-s001.zip › Supplemental Reference Sequence 1.pdf]

TAATACGACTCACTATAGGACGAAGACAAACAAACCATTATTATCATTAAAAGGCTC
AGGAGAAACTTTAACAGTAATCAAAATGTCTGTTACAGTCAAGAGAATCATTGACAA
CACAGTCGTAGTTCCAAAACTTCCTGCAAATGAGGATCCAGTGGAATACCCGGCA
GATTACTTCAGAAAATCAAAGGAGATTCCTCTTTACATCAATACTACAAAAAGTTTG
TCAGATCTAAGAGGATATGTCTACCAAGGCCTCAAATCCGGAAATGTATCAATCAT
ACATGTCAACAGCTACTTGTATGGAGCATTAAAGGACATCCGGGGTAAGTTGGATA
AAGATTGGTCAAGTTTCGGAATAAACATCGGGAAAGCAGGGGATACAATCGGAATA
TTTGACCTTGTATCCTTGAAAGCCCTGGACGGCGTACTTCCAGATGGAGTATCGGA
TGCTTCCAGAACCAGCGCAGATGACAAATGGTTGCCTTTGTATCTACTTGGCTTAT
ACAGAGTGGGCAGAACACAAATGCCTGAATACAGAAAAAAGCTCATGGATGGGCT
GACAAATCAATGCAAAATGATCAATGAACAGTTTGAACCTCTTGTGCCAGAAGGTC
GTGACATTTTTGATGTGTGGGGAAATGACAGTAATTACACAAAAATTGTCGCTGCA
GTGGACATGTTCTTCCACATGTTCAAAAAACATGAATGTGCCTCGTTCAGATACGG
AACTATTGTTTCCAGATTCAAAGATTGTGCTGCATTGGCAACATTTGGACACCTCTG
CAAAATAACCGGAATGTCTACAGAAGATGTAACGACCTGGATCTTGAACCGAGAAG
TTGCAGATGAAATGGTCCAAATGATGCTTCCAGGCCAAGAAATTGACAAGGCCGAT
TCATACATGCCTTATTTGATCGACTTTGGATTGTCTTCTAAGTCTCCATATTCTTCCG
TCAAAAACCCTGCCTTCCACTTCTGGGGGCAATTGACAGCTCTTCTGCTCAGATCC
ACCAGAGCAAGGAATGCCCGACAGCCTGATGACATTGAGTATACATCTCTTACTAC
AGCAGGTTTGTTGTACGCTTATGCAGTAGGATCCTCTGCCGACTTGGCACAACAGT
TTTGTGTTGGAGATAACAAATACACTCCAGATGATAGTACCGGAGGATTGACGACT
AATGCACCGCCACAAGGCAGAGATGTGGTCGAATGGCTCGGATGGTTTGAAGATC
AAAACAGAAAACCGACTCCTGATATGATGCAGTATGCGAAAAGAGCAGTCATGTCA
CTGCAAGGCCTAAGAGAGAAGACAATTGGCAAGTATGCTAAGTCAGAATTTGACAA
ATGACCCTATAATTCTCAGATCACCTATTATATATTATGCTACATATGAAAAAAACTA
ACAGATATCATGGATAATCTCACAAAAGTTCGTGAGTATCTCAAGTCCTATTCTCGT
CTGGATCAGGCGGTAGGAGAGATAGATGAGATCGAAGCACAACGAGCTGAAAAGT
CCAATTATGAGTTGTTCCAAGAGGATGGAGTGGAAGAGCATACTAAGCCCTCTTAT
TTTCAGGCAGCAGATGATTCTGACACAGAATCTGAACCAGAAATTGAAGACAATCA
AGGCTTGTATGCACCAGATCCAGAAGCTGAGCAAGTTGAAGGCTTTATACAGGGG
CCTTTAGATGACTATGCAGATGAGGAAGTGGATGTTGTATTTACTTCGGACTGGAA
ACAGCCTGAGCTTGAATCTGACGAGCATGGAAAGACCTTACGGTTGACATCGCCA
GAGGGTTTAAGTGGAGAGCAGAAATCCCAGTGGCTTTCGACGATTAAAGCAGTCG
TGCAAAGTGCCAAATACTGGAATCTGGCAGAGTGCACATTTGAAGCATCGGGAGA
AGGGGTCATTATGAAGGAGCGCCAGATAACTCCGGATGTATATAAGGTCACTCCA
GTGATGAACACACATCCGTCCCAATCAGAAGCAGTATCAGATGTTTGGTCTCTCTC
AAAGACATCCATGACTTTCCAACCCAAGAAAGCAAGTCTTCAGCCTCTCACCATAT
CCTTGGATGAATTGTTCTCATCTAGAGGAGAGTTCATCTCTGTCGGAGGTGACGGA
CGAATGTCTCATAAAGAGGCCATCCTGCTCGGCCTGAGATACAAAAAGTTGTACAA
TCAGGCGAGAGTCAAATATTCTCTGTAGACTATGAAAAAAAGTAACAGATATCACG

ATCTAAGTGTTATCCCAATCCATTCATCATGAGTTCCTTAAAGAAGATTCTCGGTCT
GAAGGGGAAAGGTAAGAAATCTAAGAAATTAGGGATCGCACCACCCCCTTATGAA
GAGGACACTAGCATGGAGTATGCTCCGAGCGCTCCAATTGACAAATCCTATTTTGG
AGTTGACGAGATGGACACCTATGATCCGAATCAATTAAGATATGAGAAATTCTTCTT
TACAGTGAAAATGACGGTTAGATCTAATCGTCCGTTCAGAACATACTCAGATGTGG
CAGCCGCTGTATCCCATTGGGATCACATGTACATCGGAATGGCAGGGAAACGTCC
CTTCTACAAAATCTTGGCTTTTTTGGGTTCTTCTAATCTAAAGGCCACTCCAGCGGT
ATTGGCAGATCAAGGTCAACCAGAGTATCACGCTCACTGCGAAGGCAGGGCTTAT
TTGCCACATAGGATGGGGAAGACCCCTCCCATGCTCAATGTACCAGAGCACTTCA
GAAGACCATTCAATATAGGTCTTTACAAGGGAACGATTGAGCTCACAATGACCATC
TACGATGATGAGTCACTGGAAGCAGCTCCTATGATCTGGGATCATTTCAATTCTTC
CAAATTTTCTGATTTCAGAGAGAAGGCCTTAATGTTTGGCCTGATTGTCGAGAAAAA
GGCATCTGGAGCGTGGGTCCTGGACTCTATCGGCCACTTCAAATGAGCTAGTCTA
ACTTCTAGCTTCTGAACAATCCCCGGTTTACTCAGTCTCCCCTAATTCCAGCCTCTC
GAACAACTAATATCCTGTCTTTTCTATCCCTATGAAAAAAACTAACAGAGATCGATC
TGTTTACGCGTCACTATGAAGTGCCTTTTGTACTTAGCCTTTTTATTCATTGGGGTG
AATTGCAAGTTCACCATAGTTTTTCCACACAACCAAAAAGGAAACTGGAAAAATGTT
CCTTCTAATTACCATTATTGCCCGTCAAGCTCAGATTTAAATTGGCATAATGACTTA
ATAGGCACAGCCTTACAAGTCAAAATGCCCAAGAGTCACAAGGCTATTCAAGCAGA
CGGTTGGATGTGTCATGCTTCCAAATGGGTCACTACTTGTGATTTCCGCTGGTATG
GACCGAAGTATATAACACATTCCATCCGATCCTTCACTCCATCTGTAGAACAATGCA
AGGAAAGCATTGAACAAACGAAACAAGGAACTTGGCTGAATCCAGGCTTCCCTCCT
CAAAGTTGTGGATATGCAACTGTGACGGATGCCGAAGCAGTGATTGTCCAGGTGA
CTCCTCACCATGTGCTGGTTGATGAATACACAGGAGAATGGGTTGATTCACAGTTC
ATCAACGGAAAATGCAGCAATTACATATGCCCCACTGTCCATAACTCTACAACCTG
GCATTCTGACTATAAGGTCAAAGGGCTATGTGATTCTAACCTCATTTCCATGGACAT
CACCTTCTTCTCAGAGGACGGAGAGCTATCATCCCTGGGAAAGGAGGGCACAGGG
TTCAGAAGTAACTACTTTGCTTATGAAACTGGAGGCAAGGCCTGCAAAATGCAATA
CTGCAAGCATTGGGGAGTCAGACTCCCATCAGGTGTCTGGTTCGAGATGGCTGAT
AAGGATCTCTTTGCTGCAGCCAGATTCCCTGAATGCCCAGAAGGGTCAAGTATCTC
TGCTCCATCTCAGACCTCAGTGGATGTAAGTCTAATTCAGGACGTTGAGAGGATCT
TGGATTATTCCCTCTGCCAAGAAACCTGGAGCAAAATCAGAGCGGGTCTTCCAATC
TCTCCAGTGGATCTCAGCTATCTTGCTCCTAAAAACCCAGGAACCGGTCCTGCTTT
CACCATAATCAATGGTACCCTAAAATACTTTGAGACCAGATACATCAGAGTCGATAT
TGCTGCTCCAATCCTCTCAAGAATGGTCGGAATGATCAGTGGAACTACCACAGAAA
GGGAACTGTGGGATGACTGGGCACCATATGAAGACGTGGAAATTGGACCCAATGG
AGTTCTGAGGACCAGTTCAGGATATAAGTTTCCTTTATACATGATTGGACATGGTAT
GTTGGACTCCGATCTTCATCTTAGCTCAAAGGCTCAGGTGTTCGAACATCCTCACA
TTCAAGACGCTGCTTCGCAACTTCCTGATGATGAGAGTTTATTTTTTGGTGATACTG
GGCTATCCAAAAATCCAATCGAGCTTGTAGAAGGTTGGTTCAGTAGTTGGAAAAGC

TCTATTGCCTCTTTTTTCTTTATCATAGGGTTAATCATTGGACTATTCTTGGTTCTCC
GAGTTGGTATCCATCTTTGCATTAAATTAAAGCACACCAAGAAAAGACAGATTTATA
CAGACATAGAGATGAACCGACTTGGAAAGTAACTCAAATCCTGCTAGCCAGATTCT
TCATGTTTGGACCAAATCAACTTGTGATACCATGCTCAAAGAGGCCTCAATTATATT
TGAGTTTTTAATTTTTATGAAAAAAACTAACAGCAATCATGGAAGTCCACGATTTTGA
GACCGACGAGTTCAATGATTTCAATGAAGATGACTATGCCACAAGAGAATTCCTGA
ATCCCGATGAGCGCATGACGTACTTGAATCATGCTGATTACAACCTGAATTCTCCT
CTAATTAGTGATGATATTGACAATTTAATCAGGAAATTCAATTCTCTTCCAATTCCCT
CGATGTGGGATAGTAAGAACTGGGATGGAGTTCTTGAGATGTTAACATCATGTCAA
GCCAATCCCATCTCAACATCTCAGATGCATAAATGGATGGGAAGTTGGTTAATGTC
TGATAATCATGATGCCAGTCAAGGGTATAGTTTTTTACATGAAGTGGACAAAGAGG
CAGAAATAACATTTGACGTGGTGGAGACCTTCATCCGCGGCTGGGGCAACAAACC
AATTGAATACATCAAAAAGGAAAGATGGACTGACTCATTCAAAATTCTCGCTTATTT
GTGTCAAAAGTTTTTGGACTTACACAAGTTGACATTAATCTTAAATGCTGTCTCTGA
GGTGGAATTGCTCAACTTGGCGAGGACTTTCAAAGGCAAAGTCAGAAGAAGTTCTC
ATGGAACGAACATATGCAGGATTAGGGTTCCCAGCTTGGGTCCTACTTTTATTTCA
GAAGGATGGGCTTACTTCAAGAAACTTGATATTCTAATGGACCGAAACTTTCTGTTA
ATGGTCAAAGATGTGATTATAGGGAGGATGCAAACGGTGCTATCCATGGTATGTAG
AATAGACAACCTGTTCTCAGAGCAAGACATCTTCTCCCTTCTAAATATCTACAGAAT
TGGAGATAAAATTGTGGAGAGGCAGGGAAATTTTTCTTATGACTTGATTAAAATGGT
GGAACCGATATGCAACTTGAAGCTGATGAAATTAGCAAGAGAATCAAGGCCTTTAG
TCCCACAATTCCCTCATTTTGAAAATCATATCAAGACTTCTGTTGATGAAGGGGCAA
AAATTGACCGAGGTATAAGATTCCTCCATGATCAGATAATGAGTGTGAAAACAGTG
GATCTCACACTGGTGATTTATGGATCGTTCAGACATTGGGGTCATCCTTTTATAGAT
TATTACACTGGACTAGAAAAATTACATTCCCAAGTAACCATGAAGAAAGATATTGAT
GTGTCATATGCAAAAGCACTTGCAAGTGATTTAGCTCGGATTGTTCTATTTCAACAG
TTCAATGATCATAAAAAGTGGTTCGTGAATGGAGACTTGCTCCCTCATGATCATCC
CTTTAAAAGTCATGTTAAAGAAAATACATGGCCCACAGCTGCTCAAGTTCAAGATTT
TGGAGATAAATGGCATGAACTTCCGCTGATTAAATGTTTTGAAATACCCGACTTACT
AGACCCATCGATAATATACTCTGACAAAAGTCATTCAATGAATAGGTCAGAGGTGTT
GAAACATGTCCGAATGAATCCGAACACTCCTATCCCTAGTAAAAAGGTGTTGCAGA
CTATGTTGGACACAAAGGCTACCAATTGGAAAGAATTTCTTAAAGAGATTGATGAG
AAGGGCTTAGATGATGATGATCTAATTATTGGTCTTAAAGGAAAGGAGAGGGAACT
GAAGTTGGCAGGTAGATTTTTCTCCCTAATGTCTTGGAAATTGCGAGAATACTTTGT
AATTACCGAATATTTGATAAAGACTCATTTCGTCCCTATGTTTAAAGGCCTGACAAT
GGCGGACGATCTAACTGCAGTCATTAAAAAGATGTTAGATTCCTCATCCGGCCAAG
GATTGAAGTCATATGAGGCAATTTGCATAGCCAATCACATTGATTACGAAAAATGGA
ATAACCACCAAAGGAAGTTATCAAACGGCCCAGTGTTCCGAGTTATGGGCCAGTTC
TTAGGTTATCCATCCTTAATCGAGAGAACTCATGAATTTTTTGAGAAAAGTCTTATAT
ACTACAATGGAAGACCAGACTTGATGCGTGTTCACAACAACACACTGATCAATTCA

ACCTCCCAACGAGTTTGTTGGCAAGGACAAGAGGGTGGACTGGAAGGTCTACGGC
AAAAAGGATGGAGTATCCTCAATCTACTGGTTATTCAAAGAGAGGCTAAAATCAGA
AACACTGCTGTCAAAGTCTTGGCACAAGGTGATAATCAAGTTATTTGCACACAGTAT
AAAACGAAGAAATCGAGAAACGTTGTAGAATTACAGGGTGCTCTCAATCAAATGGT
TTCTAATAATGAGAAAATTATGACTGCAATCAAAATAGGGACAGGGAAGTTAGGAC
TTTTGATAAATGACGATGAGACTATGCAATCTGCAGATTACTTGAATTATGGAAAAA
TACCGATTTTCCGTGGAGTGATTAGAGGGTTAGAGACCAAGAGATGGTCACGAGT
GACTTGTGTCACCAATGACCAAATACCCACTTGTGCTAATATAATGAGCTCAGTTTC
CACAAATGCTCTCACCGTAGCTCATTTTGCTGAGAACCCAATCAATGCCATGATAC
AGTACAATTATTTTGGGACATTTGCTAGACTCTTGTTGATGATGCATGATCCTGCTC
TTCGTCAATCATTGTATGAAGTTCAAGATAAGATACCGGGCTTGCACAGTTCTACTT
TCAAATACGCCATGTTGTATTTGGACCCTTCCATTGGAGGAGTGTCGGGCATGTCT
TTGTCCAGGTTTTTGATTAGAGCCTTCCCAGATCCCGTAACAGAAAGTCTCTCATTC
TGGAGATTCATCCATGTACATGCTCGAAGTGAGCATCTGAAGGAGATGAGTGCAGT
ATTTGGAAACCCCGAGATAGCCAAGTTTCGAATAACTCACATAGACAAGCTAGTAG
AAGATCCAACCTCTCTGAACATCGCTATGGGAATGAGTCCAGCGAACTTGTTAAAG
ACTGAGGTTAAAAAATGCTTAATCGAATCAAGACAAACCATCAGGAACCAGGTGAT
TAAGGATGCAACCATATATTTGTATCATGAAGAGGATCGGCTCAGAAGTTTCTTATG
GTCAATAAATCCTCTGTTCCCTAGATTTTTAAGTGAATTCAAATCAGGCACTTTTTTG
GGAGTCGCAGACGGGCTCATCAGTCTATTTCAAAATTCTCGTACTATTCGGAACTC
CTTTAAGAAAAAGTATCATAGGGAATTGGATGATTTGATTGTGAGGAGTGAGGTAT
CCTCTTTGACACATTTAGGGAAACTTCATTTGAGAAGGGGATCATGTAAAATGTGG
ACATGTTCAGCTACTCATGCTGACACATTAAGATACAAATCCTGGGGCCGTACAGT
TATTGGGACAACTGTACCCCATCCATTAGAAATGTTGGGTCCACAACATCGAAAAG
AGACTCCTTGTGCACCATGTAACACATCAGGGTTCAATTATGTTTCTGTGCATTGTC
CAGACGGGATCCATGACGTCTTTAGTTCACGGGGACCATTGCCTGCTTATCTAGG
GTCTAAAACATCTGAATCTACATCTATTTTGCAGCCTTGGGAAAGGGAAAGCAAAG
TCCCACTGATTAAAAGAGCTACACGTCTTAGAGATGCTATCTCTTGGTTTGTTGAAC
CCGACTCTAAACTAGCAATGACTATACTTTCTAACATCCACTCTTTAACAGGCGAAG
AATGGACCAAAAGGCAGCATGGGTTCAAAAGAACAGGGTCTGCCCTTCATAGGTTT
TCGACATCTCGGATGAGCCATGGTGGGTTCGCATCTCAGAGCACTGCAGCATTGA
CCAGGTTGATGGCAACTACAGACACCATGAGGGATCTGGGAGATCAGAATTTCGA
CTTTTTATTCCAAGCAACGTTGCTCTATGCTCAAATTACCACCACTGTTGCAAGAGA
CGGATGGATCACCAGTTGTACAGATCATTATCATATTGCCTGTAAGTCCTGTTTGAG
ACCCATAGAAGAGATCACCCTGGACTCAAGTATGGACTACACGCCCCCAGATGTAT
CCCATGTGCTGAAGACATGGAGGAATGGGGAAGGTTCGTGGGGACAAGAGATAAA
ACAGATCTATCCTTTAGAAGGGAATTGGAAGAATTTAGCACCTGCTGAGCAATCCT
ATCAAGTCGGCAGATGTATAGGTTTTCTATATGGAGACTTGGCGTATAGAAAATCTA
CTCATGCCGAGGACAGTTCTCTATTTCCTCTATCTATACAAGGTCGTATTAGAGGTC
GAGGTTTCTTAAAAGGGTTGCTAGACGGATTAATGAGAGCAAGTTGCTGCCAAGTA

ATACACCGGAGAAGTCTGGCTCATTTGAAGAGGCCGGCCAACGCAGTGTACGGAG
GTTTGATTTACTTGATTGATAAATTGAGTGTATCACCTCCATTCCTTTCTCTTACTAG
ATCAGGACCTATTAGAGACGAATTAGAAACGATTCCCCACAAGATCCCAACCTCCT
ATCCGACAAGCAACCGTGATATGGGGGTGATTGTCAGAAATTACTTCAAATACCAA
TGCCGTCTAATTGAAAAGGGAAAATACAGATCACATTATTCACAATTATGGTTATTC
TCAGATGTCTTATCCATAGACTTCATTGGACCATTCTCTATTTCCACCACCCTCTTG
CAAATCCTATACAAGCCATTTTTATCTGGGAAAGATAAGAATGAGTTGAGAGAGCT
GGCAAATCTTTCTTCATTGCTAAGATCAGGAGAGGGGTGGGAAGACATACATGTGA
AATTCTTCACCAAGGACATATTATTGTGTCCAGAGGAAATCAGACATGCTTGCAAGT
TCGGGATTGCTAAGGATAATAATAAAGACATGAGCTATCCCCCTTGGGGAAGGGAA
TCCAGAGGGACAATTACAACAATCCCTGTTTATTATACGACCACCCCTTACCCAAA
GATGCTAGAGATGCCTCCAAGAATCCAAAATCCCCTGCTGTCCGGAATCAGGTTG
GGCCAATTACCAACTGGCGCTCATTATAAAATTCGGAGTATATTACATGGAATGGG
AATCCATTACAGGGACTTCTTGAGTTGTGGAGACGGCTCCGGAGGGATGACTGCT
GCATTACTACGAGAAAATGTGCATAGCAGAGGAATATTCAATAGTCTGTTAGAATTA
TCAGGGTCAGTCATGCGAGGCGCCTCTCCTGAGCCCCCCAGTGCCCTAGAAACTT
TAGGAGGAGATAAATCGAGATGTGTAAATGGTGAAACATGTTGGGAATATCCATCT
GACTTATGTGACCCAAGGACTTGGGACTATTTCCTCCGACTCAAAGCAGGCTTGG
GGCTTCAAATTGATTTAATTGTAATGGATATGGAAGTTCGGGATTCTTCTACTAGCC
TGAAAATTGAGACGAATGTTAGAAATTATGTGCACCGGATTTTGGATGAGCAAGGA
GTTTTAATCTACAAGACTTATGGAACATATATTTGTGAGAGCGAAAAGAATGCAGTA
ACAATCCTTGGTCCCATGTTCAAGACGGTCGACTTAGTTCAAACAGAATTTAGTAGT
TCTCAAACGTCTGAAGTATATATGGTATGTAAAGGTTTGAAGAAATTAATCGATGAA
CCCAATCCCGATTGGTCTTCCATCAATGAATCCTGGAAAAACCTGTACGCATTCCA
GTCATCAGAACAGGAATTTGCCAGAGCAAAGAAGGTTAGTACATACTTTACCTTGA
CAGGTATTCCCTCCCAATTCATTCCTGATCCTTTTGTAAACATTGAGACTATGCTAC
AAATATTCGGAGTACCCACGGGTGTGTCTCATGCGGCTGCCTTAAAATCATCTGAT
AGACCTGCAGATTTATTGACCATTAGCCTTTTTTATATGGCGATTATATCGTATTATA
ACATCAATCATATCAGAGTAGGACCGATACCTCCGAACCCCCCATCAGATGGAATT
GCACAAAATGTGGGGATCGCTATAACTGGTATAAGCTTTTGGCTGAGTTTGATGGA
GAAAGACATTCCACTATATCAACAGTGTTTAGCAGTTATCCAGCAATCATTCCCGAT
TAGGTGGGAGGCTGTTTCAGTAAAAGGAGGATACAAGCAGAAGTGGAGTACTAGA
GGTGATGGGCTCCCAAAAGATACCCGAATTTCAGACTCCTTGGCCCCAATCGGGA
ACTGGATCAGATCTCTGGAATTGGTCCGAAACCAAGTTCGTCTAAATCCATTCAAT
GAGATCTTGTTCAATCAGCTATGTCGTACAGTGGATAATCATTTGAAATGGTCAAAT
TTGCGAAGAAACACAGGAATGATTGAATGGATCAATAGACGAATTTCAAAAGAAGA
CCGGTCTATACTGATGTTGAAGAGTGACCTACACGAGGAAAACTCTTGGAGAGATT
AAAAAATCATGAGGAGACTCCAAACTTTAAGTATGAAAAAAACTTTGATCCTTAAGA
CCCTCTTGTGGTTTTTATTTTTTATCTGGTTTTGTGGTCTTCGTGGGTCGGCATGGC
ATCTCCACCTCCTCGCGGTCCGACCTGGGCATCCGAAGGAGGACGTCGTCCACTC

GGATGGCTAAGGGAGGGGCCCCCGCGGGGCTGCTAACAAAGCCCGAAAGGAAG
CTGAGTTGGCTGCTGCCACCGCTGAGCAATAACTAGCATAACCCCTTGGGGCCTC
TAAACGGGTCTTGAGGGGTTTTTTGCTGAAAGGAGGAACTATATCCGGATCGAGAC
CTCGATACTAGTGCGGTGGAGCTCCAGCTTTTGTTCCCTTTAGTGAGGGTTAATTT
CGAGCTTGGCGTAATCATGGTCATAGCTGTTTCCTGTGTGAAATTGTTATCCGCTC
ACAATTCCACACAACATACGAGCCGGAAGCATAAAGTGTAAAGCCTGGGGTGCCT
AATGAGTGAGCTAACTCACATTAATTGCGTTGCGCTCACTGCCCGCTTTCCAGTCG
GGAAACCTGTCGTGCCAGCTGCATTAATGAATCGGCCAACGCGCGGGGAGAGGC
GGTTTGCGTATTGGGCGCTCTTCCGCTTCCTCGCTCACTGACTCGCTGCGCTCGG
TCGTTCGGCTGCGGCGAGCGGTATCAGCTCACTCAAAGGCGGTAATACGGTTATC
CACAGAATCAGGGGATAACGCAGGAAAGAACATGTGAGCAAAAGGCCAGCAAAAG
GCCAGGAACCGTAAAAAGGCCGCGTTGCTGGCGTTTTTCCATAGGCTCCGCCCCC
CTGACGAGCATCACAAAAATCGACGCTCAAGTCAGAGGTGGCGAAACCCGACAGG
ACTATAAAGATACCAGGCGTTTCCCCCTGGAAGCTCCCTCGTGCGCTCTCCTGTTC
CGACCCTGCCGCTTACCGGATACCTGTCCGCCTTTCTCCCTTCGGGAAGCGTGGC
GCTTTCTCATAGCTCACGCTGTAGGTATCTCAGTTCGGTGTAGGTCGTTCGCTCCA
AGCTGGGCTGTGTGCACGAACCCCCCGTTCAGCCCGACCGCTGCGCCTTATCCG
GTAACTATCGTCTTGAGTCCAACCCGGTAAGACACGACTTATCGCCACTGGCAGCA
GCCACTGGTAACAGGATTAGCAGAGCGAGGTATGTAGGCGGTGCTACAGAGTTCT
TGAAGTGGTGGCCTAACTACGGCTACACTAGAAGAACAGTATTTGGTATCTGCGCT
CTGCTGAAGCCAGTTACCTTCGGAAAAAGAGTTGGTAGCTCTTGATCCGGCAAACA
AACCACCGCTGGTAGCGGTGGTTTTTTTGTTTGCAAGCAGCAGATTACGCGCAGAA
AAAAAGGATCTCAAGAAGATCCTTTGATCTTTTCTACGGGGTCTGACGCTCAGTGG
AACGAAAACTCACGTTAAGGGATTTTGGTCATGAGATTATCAAAAAGGATCTTCACC
TAGATCCTTTTAAATTAAAAATGAAGTTTTAAATCAATCTAAAGTATATATGAGTAAA
CTTGGTCTGACAGTTACCAATGCTTAATCAGTGAGGCACCTATCTCAGCGATCTGT
CTATTTCGTTCATCCATAGTTGCCTGACTCCCCGTCGTGTAGATAACTACGATACG
GGAGGGCTTACCATCTGGCCCCAGTGCTGCAATGATACCGCGAGACCCACGCTCA
CCGGCTCCAGATTTATCAGCAATAAACCAGCCAGCCGGAAGGGCCGAGCGCAGAA
GTGGTCCTGCAACTTTATCCGCCTCCATCCAGTCTATTAATTGTTGCCGGGAAGCT
AGAGTAAGTAGTTCGCCAGTTAATAGTTTGCGCAACGTTGTTGCCATTGCTACAGG
CATCGTGGTGTCACGCTCGTCGTTTGGTATGGCTTCATTCAGCTCCGGTTCCCAAC
GATCAAGGCGAGTTACATGATCCCCCATGTTGTGCAAAAAAGCGGTTAGCTCCTTC
GGTCCTCCGATCGTTGTCAGAAGTAAGTTGGCCGCAGTGTTATCACTCATGGTTAT
GGCAGCACTGCATAATTCTCTTACTGTCATGCCATCCGTAAGATGCTTTTCTGTGAC
TGGTGAGTACTCAACCAAGTCATTCTGAGAATAGTGTATGCGGCGACCGAGTTGCT
CTTGCCCGGCGTCAATACGGGATAATACCGCGCCACATAGCAGAACTTTAAAAGT
GCTCATCATTGGAAAACGTTCTTCGGGGCGAAAACTCTCAAGGATCTTACCGCTGT
TGAGATCCAGTTCGATGTAACCCACTCGTGCACCCAACTGATCTTCAGCATCTTTTA
CTTTCACCAGCGTTTCTGGGTGAGCAAAAACAGGAAGGCAAAATGCCGCAAAAAA

GGGAATAAGGGCGACACGGAAATGTTGAATACTCATACTCTTCCTTTTTCAATATTA
TTGAAGCATTTATCAGGGTTATTGTCTCATGAGCGGATACATATTTGAATGTATTTA
GAAAAATAAACAAATAGGGGTTCCGCGCACATTTCCCCGAAAAGTGCCACCTAAAT
TGTAAGCGTTAATATTTTGTTAAAATTCGCGTTAAATTTTTGTTAAATCAGCTCATTTT
TTAACCAATAGGCCGAAATCGGCAAAATCCCTTATAAATCAAAAGAATAGACCGAG
ATAGGGTTGAGTGTTGTTCCAGTTTGGAACAAGAGTCCACTATTAAAGAACGTGGA
CTCCAACGTCAAAGGGCGAAAAACCGTCTATCAGGGCGATGGCCCACTACGTGAA
CCATCACCCTAATCAAGTTTTTTGGGGTCGAGGTGCCGTAAAGCACTAAATCGGAA
CCCTAAAGGGAGCCCCCGATTTAGAGCTTGACGGGGAAAGCCGGCGAACGTGGC
GAGAAAGGAAGGGAAGAAAGCGAAAGGAGCGGGCGCTAGGGCGCTGGCAAGTGT
AGCGGTCACGCTGCGCGTAACCACCACACCCGCCGCGCTTAATGCGCCGCTACA
GGGCGCGTCCCATTCGCCATTCAGGCTGCGCAACTGTTGGGAAGGGCGATCGGT
GCGGGCCTCTTCGCTATTACGCCAGCTGGCGAAAGGGGGATGTGCTGCAAGGCG
ATTAAGTTGGGTAACGCCAGGGTTTTCCCAGTCACGACGTTGTAAAACGACGGCC
AGTGAATTG
