# Supplementary material for: Leveraging Synthetic Virology for the Rapid Engineering of Vesicular Stomatitis Virus (VSV)
Source: Viruses. 2024 Oct 21;16(10):1641. doi: 10.3390/v16101641 (PMC11512388; doi:10.3390/v16101641)
Supplement: Supplementary file 1 [file viruses-16-01641-s001.zip › Supplemental Reference Sequence 2.pdf]

TAATACGACTCACTATAGGACGAAGACAAACAAACCATTATTATCATTAAAAGGCTC
AGGAGAAACTTTAACAGTAATCAAAATGTCTGTTACAGTCAAGAGAATCATTGACAA
CACAGTCATAGTTCCAAAACTTCCTGCAAATGAGGATCCAGTGGAATACCCGGCAG
ATTACTTCAGAAAATCAAAGGAGATTCCTCTTTACATCAATACTACAAAAAGTTTGTC
AGATCTAAGAGGATATGTCTACCAAGGCCTCAAATCCGGAAATGTATCAATCATAC
ATGTCAACAGCTACTTGTATGGAGCATTAAAGGACATCCGGGGTAAGTTGGATAAA
GATTGGTCAAGTTTCGGAATAAACATCGGGAAAGCAGGGGATACAATCGGAATATT
TGACCTTGTATCCTTGAAAGCCCTGGACGGCGTACTTCCAGATGGAGTATCGGATG
CTTCCAGAACCAGCGCAGATGACAAATGGTTGCCTTTGTATCTACTTGGCTTATAC
AGAGTGGGCAGAACACAAATGCCTGAATACAGAAAAAAGCTCATGGATGGGCTGA
CAAATCAATGCAAAATGATCAATGAACAGTTTGAACCTCTTGTGCCAGAAGGTCGT
GACATTTTTGATGTGTGGGGAAATGACAGTAATTACACAAAAATTGTCGCTGCAGT
GGACATGTTCTTCCACATGTTCAAAAAACATGAATGTGCCTCGTTCAGATACGGAA
CTATTGTTTCCAGATTCAAAGATTGTGCTGCATTGGCAACATTTGGACACCTCTGCA
AAATAACCGGAATGTCTACAGAAGATGTAACGACCTGGATCTTGAACCGAGAAGTT
GCAGATGAAATGGTCCAAATGATGCTTCCAGGCCAAGAAATTGACAAGGCCGATTC
ATACATGCCTTATTTGATCGACTTTGGATTGTCTTCTAAGTCTCCATATTCTTCCGTC
AAAAACCCTGCCTTCCACTTCTGGGGGCAATTGACAGCTCTTCTGCTCAGATCCAC
CAGAGCAAGGAATGCCCGACAGCCTGATGACATTGAGTATACATCTCTTACTACAG
CAGGTTTGTTGTACGCTTATGCAGTAGGATCCTCTGCCGACTTGGCACAACAGTTT
TGTGTTGGAGATAACAAATACACTCCAGATGATAGTACCGGAGGATTGACGACTAA
TGCACCGCCACAAGGCAGAGATGTGGTCGAATGGCTCGGATGGTTTGAAGATCAA
AACAGAAAACCGACTCCTGATATGATGCAGTATGCGAAAAGAGCAGTCATGTCACT
GCAAGGCCTAAGAGAGAAGACAATTGGCAAGTATGCTAAGTCAGAATTTGACAAAT
GACCCTATAATTCTCAGATCACCTATTATATATTATGCTACATATGAAAAAAACTAAC
AGATATCATGGATAATCTCACAAAAGTTCGTGAGTATCTCAAGTCCTATTCTCGTCT
GGATCAGGCGGTAGGAGAGATAGATGAGATCGAAGCACAACGAGCTGAAAAGTCC
AATTATGAGTTGTTCCAAGAGGATGGAGTGGAAGAGCATACTAAGCCCTCTTATTT
TCAGGCAGCAGATGATTCTGACACAGAATCTGAACCAGAAATTGAAGACAATCAAG
GTTTGTATGCACAGGATCCAGAAGCTGAGCAAGTTGAAGGCTTTATACAGGGGCC
TTTAGATGACTATGCAGATGAGGAAGTGGATGTTGTATTTACTTCGGACTGGAAAC
CACCTGAGCTTGAATCTGACGAGCATGGAAAGACCTTACGGTTGACATCGCCAGA
GGGTTTAAGTGGAGAGCAGAAATCCCAGTGGCTTTCGACGATTAAAGCAGTCGTG
CAAAGTGCCAAATACTGGAATCTGGCAGAGTGCACATTTGAAGCATCGGGAGAAG
GGGTCATTATGAAGGAGCGCCAGATAACTCCGGATGTATATAAGGTCACTCCAGT
GATGAACACACATCCGTCCCAATCAGAAGCAGTATCAGATGTTTGGTCTCTCTCAA
AGACATCCATGACTTTCCAACCCAAGAAAGCAAGTCTTCAGCCTCTCACCATATCC
TTGGATGAATTGTTCTCATCTAGAGGAGAGTTCATCTCTGTCGGAGGTGACGGACG
AATGTCTCATAAAGAGGCCATCCTGCTCGGCCTGAGATACAAAAAGTTGTACAATC
AGGCGAGAGTCAAATATTCTCTGTAGACTATGAAAAAAAGTAACAGATATCACGAT

CTAAGTGTTATCCCAATCCATTCATCATGAGTTCCTTAAAGAAGATTCTCGGTCTGA
AGGGGAAAGGTAAGAAATCTAAGAAATTAGGGATCGCACCACCCCCTTATGAAGA
GGACACTAGCATGGAGTATGCTCCGAGCGCTCCAATTGACAAATCCTATTTTGGAG
TTGACGAGATGGACACCTATGATCCGAATCAATTAAGATATGAGAAATTCTTCTTTA
CAGTGAAAATGACGGTTAGATCTAATCGTCCGTTCAGAACATACTCAGATGTGGCA
GCCGCTGTATCCCATTGGGATCACATGTACATCGGAATGGCAGGGAAACGTCCCT
TCTACAAAATCTTGGCTTTTTTGGGTTCTTCTAATCTAAAGGCCACTCCAGCGGTAT
TGGCAGATCAAGGTCAACCAGAGTATCACACTCACTGCGAAGGCAGGGCTTATTT
GCCACATAGGATGGGGAAGACCCCTCCCATGCTCAATGTACCAGAGCACTTCAGA
AGACCATTCAATATAGGTCTTTACAAGGGAACGATTGAGCTCACAATGACCATCTA
CGATGATGAGTCACTGGAAGCAGCTCCTATGATCTGGGATCATTTCAATTCTTCCA
AATTTTCTGATTTCAGAGAGAAGGCCTTAATGTTTGGCCTGATTGTCGAGAAAAAG
GCATCTGGAGCGTGGGTCCTGGATTCTATCAGCCACTTCAAATGAGCTAGTCTAAC
TTCTAGCTTCTGAACAATCCCCGGTTTACTCAGTCTCTCCTAATTCCAGCCTCTCGA
ACAACTAATATCCTGTCTTTTCTATCCCTATGAAAAAAACTAACAGAGATCGATCTG
TTTACGCGTCACTATGAAGTGCCTTTTGTACTTAGCCTTTTTATTCATTGGGGTGAA
TTGCAAGTTCACCATAGTTTTTCCACACAACCAAAAAGGAAACTGGAAAAATGTTCC
TTCTAATTACCATTATTGCCCGTCAAGCTCAGATTTAAATTGGCATAATGACTTAATA
GGCACAGCCATACAAGTCAAAATGCCCAAGAGTCACAAGGCTATTCAAGCAGACG
GTTGGATGTGTCATGCTTCCAAATGGGTCACTACTTGTGATTTCCGCTGGTATGGA
CCGAAGTATATAACACAGTCCATCCGATCCTTCACTCCATCTGTAGAACAATGCAA
GGAAAGCATTGAACAAACGAAACAAGGAACTTGGCTGAATCCAGGCTTCCCTCCTC
AAAGTTGTGGATATGCAACTGTGACGGATGCCGAAGCAGTGATTGTCCAGGTGAC
TCCTCACCATGTGCTGGTTGATGAATACACAGGAGAATGGGTTGATTCACAGTTCA
TCAACGGAAAATGCAGCAATTACATATGCCCCACTGTCCATAACTCTACAACCTGG
CATTCTGACTATAAGGTCAAAGGGCTATGTGATTCTAACCTCATTTCCATGGACATC
ACCTTCTTCTCAGAGGACGGAGAGCTATCATCCCTGGGAAAGGAGGGCACAGGGT
TCAGAAGTAACTACTTTGCTTATGAAACTGGAGGCAAGGCCTGCAAAATGCAATAC
TGCAAGCATTGGGGAGTCAGACTCCCATCAGGTGTCTGGTTCGAGATGGCTGATA
AGGATCTCTTTGCTGCAGCCAGATTCCCTGAATGCCCAGAAGGGTCAAGTATCTCT
GCTCCATCTCAGACCTCAGTGGATGTAAGTCTAATTCAGGACGTTGAGAGGATCTT
GGATTATTCCCTCTGCCAAGAAACCTGGAGCAAAATCAGAGCGGGTCTTCCAATCT
CTCCAGTGGATCTCAGCTATCTTGCTCCTAAAAACCCAGGAACCGGTCCTGCTTTC
ACCATAATCAATGGTACCCTAAAATACTTTGAGACCAGATACATCAGAGTCGATATT
GCTGCTCCAATCCTCTCAAGAATGGTCGGAATGATCAGTGGAACTACCACAGAAAG
GGAACTGTGGGATGACTGGGCACCATATGAAGACGTGGAAATTGGACCCAATGGA
GTTCTGAGGACCAGTTCAGGATATAAGTTTCCTTTATACATGATTGGACATGGTATG
TTGGACTCCGATCTTCATCTTAGCTCAAAGGCTCAGGTGTTCGAACATCCTCACATT
CAAGACGCTGCTTCGCAACTTCCTGATGATGAGAGTTTATTTTTTGGTGATACTGG
GCTATCCAAAAATCCAATCGAGCTTGTAGAAGGTTGGTTCAGTAGTTGGAAAAGCT

CTATTGCCTCTTTTTTCTTTATCATAGGGTTAATCATTGGACTATTCTTGGTTCTCCG
AGTTGGTATCCATCTTTGCATTAAATTAAAGCACACCAAGAAAAGACAGATTTATAC
AGACATAGAGATGAACCGACTTGGAAAGTAACTCAAATCCTGCTAGCCAGATTCTT
CATGTTTGGACCAAATCAACTTGTGATACCATGCTCAAAGAGGCCTCAATTATATTT
GAGTTTTTAATTTTTATGAAAAAAACTAACAGCAATCATGGAAGTCCACGATTTTGA
GACCGACGAGTTCAATGATTTCAATGAAGATGACTATGCCACAAGAGAATTCCTGA
ATCCCGATGAGCGCATGACGTACTTGAATCATGCTGATTACAACCTGAATTCTCCT
CTAATTAGTGATGATATTGACAATTTAATCAGGAAATTCAATTCTCTTCCAATTCCCT
CGATGTGGGATAGTAAGAACTGGGATGGAGTTCTTGAGATGTTAACATCATGTCAA
GCCAATCCCATCTCAACATCTCAGATGCATAAATGGATGGGAAGTTGGTTAATGTC
TGATAATCATGATGCCAGTCAAGGGTATAGTTTTTTACATGAAGTGGACAAAGAGG
CAGAAATAACATTTGACGTGGTGGAGACCTTCATCCGCGGCTGGGGCAACAAACC
AATTGAATACATCAAAAAGGAAAGATGGACTGACTCATTCAAAATTCTCGCTTATTT
GTGTCAAAAGTTTTTGGACTTACACAAGTTGACATTAATCTTAAATGCTGTCTCTGA
GGTGGAATTGCTCAACTTGGCGAGGACTTTCAAAGGCAAAGTCAGAAGAAGTTCTC
ATGGAACGAACATATGCAGGATTAGGGTTCCCAGCTTGGGTCCTACTTTTATTTCA
GAAGGATGGGCTTACTTCAAGAAACTTGATATTCTAATGGACCGAAACTTTCTGTTA
ATGGTCAAAGATGTGATTATAGGGAGGATGCAAACGGTGCTATCCATGGTATGTAG
AATAGACAACCTGTTCTCAGAGCAAGACATCTTCTCCCTTCTAAATATCTACAGAAT
TGGAGATAAAATTGTGGAGAGGCAGGGAAATTTTTCTTATGACTTGATTAAAATGGT
GGAACCGATATGCAACTTGAAGCTGATGAAATTAGCAAGAGAATCAAGGCCTTTAG
TCCCACAATTCCCTCATTTTGAAAATCATATCAAGACTTCTGTTGATGAAGGGGCAA
AAATTGACCGAGGTATAAGATTCCTCCATGATCAGATAATGAGTGTGAAAACAGTG
GATCTCACACTGGTGATTTATGGATCGTTCAGACATTGGGGTCATCCTTTTATAGAT
TATTACACTGGACTAGAAAAATTACATTCCCAAGTAACCATGAAGAAAGATATTGAT
GTGTCATATGCAAAAGCACTTGCAAGTGATTTAGCTCGGATTGTTCTATTTCAACAG
TTCAATGATCATAAAAAGTGGTTCGTGAATGGAGACTTGCTCCCTCATGATCATCC
CTTTAAAAGTCATGTTAAAGAAAATACATGGCCCACAGCTGCTCAAGTTCAAGATTT
TGGAGATAAATGGCATGAACTTCCGCTGATTAAATGTTTTGAAATACCCGACTTACT
AGACCCATCGATAATATACTCTGACAAAAGTCATTCAATGAATAGGTCAGAGGTGTT
GAAACATGTCCGAATGAATCCGAACACTCCTATCCCTAGTAAAAAGGTGTTGCAGA
CTATGTTGGACACAAAGGCTACCAATTGGAAAGAATTTCTTAAAGAGATTGATGAG
AAGGGCTTAGATGATGATGATCTAATTATTGGTCTTAAAGGAAAGGAGAGGGAACT
GAAGTTGGCAGGTAGATTTTTCTCCCTAATGTCTTGGAAATTGCGAGAATACTTTGT
AATTACCGAATATTTGATAAAGACTCATTTCGTCCCTATGTTTAAAGGCCTGACAAT
GGCGGACGATCTAACTGCAGTCATTAAAAAGATGTTAGATTCCTCATCCGGCCAAG
GATTGAAGTCATATGAGGCAATTTGCATAGCCAATCACATTGATTACGAAAAATGGA
ATAACCACCAAAGGAAGTTATCAAACGGCCCAGTGTTCCGAGTTATGGGCCAGTTC
TTAGGTTATCCATCCTTAATCGAGAGAACTCATGAATTTTTTGAGAAAAGTCTTATAT
ACTACAATGGAAGACCAGACTTGATGCGTGTTCACAACAACACACTGATCAATTCA

ACCTCCCAACGAGTTTGTTGGCAAGGACAAGAGGGTGGACTGGAAGGTCTACGGC
AAAAAGGATGGACTATCCTCAATCTACTGGTTATTCAAAGAGAGGCTAAAATCAGA
AACACTGCTGTCAAAGTCTTGGCACAAGGTGATAATCAAGTTATTTGCACACAGTAT
AAAACGAAGAAATCGAGAAACGTTGTAGAATTACAGGGTGCTCTCAATCAAATGGT
TTCTAATAATGAGAAAATTATGACTGCAATCAAAATAGGGACAGGGAAGTTAGGAC
TTTTGATAAATGACGATGAGACTATGCAATCTGCAGATTACTTGAATTATGGAAAAA
TACCGATTTTCCGTGGAGTGATTAGAGGGTTAGAGACCAAGAGATGGTCACGAGT
GACTTGTGTCACCAATGACCAAATACCCACTTGTGCTAATATAATGAGCTCAGTTTC
CACAAATGCTCTCACCGTAGCTCATTTTGCTGAGAACCCAATCAATGCCATGATAC
AGTACAATTATTTTGGGACATTTGCTAGACTCTTGTTGATGATGCATGATCCTGCTC
TTCGTCAATCATTGTATGAAGTTCAAGATAAGATACCGGGCTTGCACAGTTCTACTT
TCAAATACGCCATGTTGTATTTGGACCCTTCCATTGGAGGAGTGTCGGGCATGTCT
TTGTCCAGGTTTTTGATTAGAGCCTTCCCAGATCCCGTAACAGAAAGTCTCTCATTC
TGGAGATTCATCCATGTACATGCTCGAAGTGAGCATCTGAAGGAGATGAGTGCAGT
ATTTGGAAACCCCGAGATAGCCAAGTTTCGAATAACTCACATAGACAAGCTAGTAG
AAGATCCAACCTCTCTGAACATCGCTATGGGAATGAGTCCAGCGAACTTGTTAAAG
ACTGAGGTTAAAAAATGCTTAATCGAATCAAGACAAACCATCAGGAACCAGGTGAT
TAAGGATGCAACCATATATTTGTATCATGAAGAGGATCGGCTCAGAAGTTTCTTATG
GTCAATAAATCCTCTGTTCCCTAGATTTTTAAGTGAATTCAAATCAGGCACTTTTTTG
GGAGTCGCAGACGGGCTCATCAGTCTATTTCAAAATTCTCGTACTATTCGGAACTC
CTTTAAGAAAAAGTATCATAGGGAATTGGATGATTTGATTGTGAGGAGTGAGGTAT
CCTCTTTGACACATTTAGGGAAACTTCATTTGAGAAGGGGATCATGTAAAATGTGG
ACATGTTCAGCTACTCATGCTGACACATTAAGATACAAATCCTGGGGCCGTACAGT
TATTGGGACAACTGTACCCCATCCATTAGAAATGTTGGGTCCACAACATCGAAAAG
AGACTCCTTGTGCACCATGTAACACATCAGGGTTCAATTATGTTTCTGTGCATTGTC
CAGACGGGATCCATGACGTCTTTAGTTCACGGGGACCATTGCCTGCTTATCTAGG
GTCTAAAACATCTGAATCTACATCTATTTTGCAGCCTTGGGAAAGGGAAAGCAAAG
TCCCACTGATTAAAAGAGCTACACGTCTTAGAGATGCTATCTCTTGGTTTGTTGAAC
CCGACTCTAAACTAGCAATGACTATACTTTCTAACATCCACTCTTTAACAGGCGAAG
AATGGACCAAAAGGCAGCATGGGTTCAAAAGAACAGGGTCTGCCCTTCATAGGTTT
TCGACATCTCGGATGAGCCATGGTGGGTTCGCATCTCAGAGCACTGCAGCATTGA
CCAGGTTGATGGCAACTACAGACACCATGAGGGATCTGGGAGATCAGAATTTCGA
CTTTTTATTCCAAGCAACGTTGCTCTATGCTCAAATTACCACCACTGTTGCAAGAGA
CGGATGGATCACCAGTTGTACAGATCATTATCATATTGCCTGTAAGTCCTGTTTGAG
ACCCATAGAAGAGATCACCCTGGACTCAAGTATGGACTACACGCCCCCAGATGTAT
CCCATGTGCTGAAGACATGGAGGAATGGGGAAGGTTCGTGGGGACAAGAGATAAA
ACAGATCTATCCTTTAGAAGGGAATTGGAAGAATTTAGCACCTGCTGAGCAATCCT
ATCAAGTCGGCAGATGTATAGGTTTTCTATATGGAGACTTGGCGTATAGAAAATCTA
CTCATGCCGAGGACAGTTCTCTATTTCCTCTATCTATACAAGGTCGTATTAGAGGTC
GAGGTTTCTTAAAAGGGTTGCTAGACGGATTAATGAGAGCAAGTTGCTGCCAAGTA

ATACACCGGAGAAGTCTGGCTCATTTGAAGAGGCCGGCCAACGCAGTGTACGGAG
GTTTGATTTACTTGATTGATAAATTGAGTGTATCACCTCCATTCCTTTCTCTTACTAG
ATCAGGACCTATTAGAGACGAATTAGAAACGATTCCCCACAAGATCCCAACCTCCT
ATCCGACAAGCAACCGTGATATGGGGGTGATTGTCAGAAATTACTTCAAATACCAA
TGCCGTCTAATTGAAAAGGGAAAATACAGATCACATTATTCACAATTATGGTTATTC
TCAGATGTCTTATCCATAGACTTCATTGGACCATTCTCTATTTCCACCACCCTCTTG
CAAATCCTATACAAGCCATTTTTATCTGGGAAAGATAAGAATGAGTTGAGAGAGCT
GGCAAATCTTTCTTCATTGCTAAGATCAGGAGAGGGGTGGGAAGACATACATGTGA
AATTCTTCACCAAGGACATATTATTGTGTCCAGAGGAAATCAGACATGCTTGCAAGT
TCGGGATTGCTAAGGATAATAATAAAGACATGAGCTATCCCCCTTGGGGAAGGGAA
TCCAGAGGGACAATTACAACAATCCCTGTTTATTATACGACCACCCCTTACCCAAA
GATGCTAGAGATGCCTCCAAGAATCCAAAATCCCCTGCTGTCCGGAATCAGGTTG
GGCCAATTACCAACTGGCGCTCATTATAAAATTCGGAGTATATTACATGGAATGGG
AATCCATTACAGGGACTTCTTGAGTTGTGGAGACGGCTCCGGAGGGATGACTGCT
GCATTACTACGAGAAAATGTGCATAGCAGAGGAATATTCAATAGTCTGTTAGAATTA
TCAGGGTCAGTCATGCGAGGCGCCTCTCCTGAGCCCCCCAGTGCCCTAGAAACTT
TAGGAGGAGATAAATCGAGATGTGTAAATGGTGAAACATGTTGGGAATATCCATCT
GACTTATGTGACCCAAGGACTTGGGACTATTTCCTCCGACTCAAAGCAGGCTTGG
GGCTTCAAATTGATTTAATTGTAATGGATATGGAAGTTCGGGATTCTTCTACTAGCC
TGAAAATTGAGACGAATGTTAGAAATTATGTGCACCGGATTTTGGATGAGCAAGGA
GTTTTAATCTACAAGACTTATGGAACATATATTTGTGAGAGCGAAAAGAATGCAGTA
ACAATCCTTGGTCCCATGTTCAAGACGGTCGACTTAGTTCAAACAGAATTTAGTAGT
TCTCAAACGTCTGAAGTATATATGGTATGTAAAGGTTTGAAGAAATTAATCGATGAA
CCCAATCCCGATTGGTCTTCCATCAATGAATCCTGGAAAAACCTGTACGCATTCCA
GTCATCAGAACAGGAATTTGCCAGAGCAAAGAAGGTTAGTACATACTTTACCTTGA
CAGGTATTCCCTCCCAATTCATTCCTGATCCTTTTGTAAACATTGAGACTATGCTAC
AAATATTCGGAGTACCCACGGGTGTGTCTCATGCGGCTGCCTTAAAATCATCTGAT
AGACCTGCAGATTTATTGACCATTAGCCTTTTTTATATGGCGATTATATCGTATTATA
ACATCAATCATATCAGAGTAGGACCGATACCTCCGAACCCCCCATCAGATGGAATT
GCACAAAATGTGGGGATCGCTATAACTGGTATAAGCTTTTGGCTGAGTTTGATGGA
GAAAGACATTCCACTATATCAACAGTGTTTAGCAGTTATCCAGCAATCATTCCCGAT
TAGGTGGGAGGCTGTTTCAGTAAAAGGAGGATACAAGCAGAAGTGGAGTACTAGA
GGTGATGGGCTCCCAAAAGATACCCGAACTTCAGACTCCTTGGCCCCAATCGGGA
ACTGGATCAGATCTCTGGAATTGGTCCGAAACCAAGTTCGTCTAAATCCATTCAAT
GAGATCTTGTTCAATCAGCTATGTCGTACAGTGGATAATCATTTGAAATGGTCAAAT
TTGCGAAGAAACACAGGAATGATTGAATGGATCAATAGACGAATTTCAAAAGAAGA
CCGGTCTATACTGATGTTGAAGAGTGACCTACACGAGGAAAACTCTTGGAGAGATT
AAAAAATCATGAGGAGACTCCAAACTTTAAGTATGAAAAAAACTTTGATCCTTAAGA
CCCTCTTGTGGTTTTTATTTTTTATCTGGTTTTGTGGTCTTCGTGGGTCGGCATGGC
ATCTCCACCTCCTCGCGGTCCGACCTGGGCATCCGAAGGAGGACGTCGTCCACTC

GGATGGCTAAGGGAGGGGCCCCCGCGGGGCTGCTAACAAAGCCCGAAAGGAAG
CTGAGTTGGCTGCTGCCACCGCTGAGCAATAACTAGCATAACCCCTTGGGGCCTC
TAAACGGGTCTTGAGGGGTTTTTTGCTGAAAGGAGGAACTATATCCGGATCGAGAC
CTCGATACTAGTGAGCTCCAGCTTTTGTTCCCTTTAGTGAGGGTTAATTTCGAGCTT
GGCGTAATCATGGTCATAGCTGTTTCCTGTGTGAAATTGTTATCCGCTCACAATTCC
ACACAACATACGAGCCGGAAGCATAAAGTGTAAAGCCTGGGGTGCCTAATGAGTG
AGCTAACTCACATTAATTGCGTTGCGCTCACTGCCCGCTTTCCAGTCGGGAAACCT
GTCGTGCCAGCTGCATTAATGAATCGGCCAACGCGCGGGGAGAGGCGGTTTGCG
TATTGGGCGCTCTTCCGCTTCCTCGCTCACTGACTCGCTGCGCTCGGTCGTTCGG
CTGCGGCGAGCGGTATCAGCTCACTCAAAGGCGGTAATACGGTTATCCACAGAAT
CAGGGGATAACGCAGGAAAGAACATGTGAGCAAAAGGCCAGCAAAAGGCCAGGA
ACCGTAAAAAGGCCGCGTTGCTGGCGTTTTTCCATAGGCTCCGCCCCCCTGACGA
GCATCACAAAAATCGACGCTCAAGTCAGAGGTGGCGAAACCCGACAGGACTATAA
AGATACCAGGCGTTTCCCCCTGGAAGCTCCCTCGTGCGCTCTCCTGTTCCGACCC
TGCCGCTTACCGGATACCTGTCCGCCTTTCTCCCTTCGGGAAGCGTGGCGCTTTC
TCATAGCTCACGCTGTAGGTATCTCAGTTCGGTGTAGGTCGTTCGCTCCAAGCTGG
GCTGTGTGCACGAACCCCCCGTTCAGCCCGACCGCTGCGCCTTATCCGGTAACTA
TCGTCTTGAGTCCAACCCGGTAAGACACGACTTATCGCCACTGGCAGCAGCCACT
GGTAACAGGATTAGCAGAGCGAGGTATGTAGGCGGTGCTACAGAGTTCTTGAAGT
GGTGGCCTAACTACGGCTACACTAGAAGGACAGTATTTGGTATCTGCGCTCTGCTG
AAGCCAGTTACCTTCGGAAAAAGAGTTGGTAGCTCTTGATCCGGCAAACAAACCAC
CGCTGGTAGCGGTGGTTTTTTTGTTTGCAAGCAGCAGATTACGCGCAGAAAAAAAG
GATCTCAAGAAGATCCTTTGATCTTTTCTACGGGGTCTGACGCTCAGTGGAACGAA
AACTCACGTTAAGGGATTTTGGTCATGAGATTATCAAAAAGGATCTTCACCTAGATC
CTTTTAAATTAAAAATGAAGTTTTAAATCAATCTAAAGTATATATGAGTAAACTTGGT
CTGACAGTTACCAATGCTTAATCAGTGAGGCACCTATCTCAGCGATCTGTCTATTTC
GTTCATCCATAGTTGCCTGACTCCCCGTCGTGTAGATAACTACGATACGGGAGGG
CTTACCATCTGGCCCCAGTGCTGCAATGATACCGCGAGACCCACGCTCACCGGCT
CCAGATTTATCAGCAATAAACCAGCCAGCCGGAAGGGCCGAGCGCAGAAGTGGTC
CTGCAACTTTATCCGCCTCCATCCAGTCTATTAATTGTTGCCGGGAAGCTAGAGTA
AGTAGTTCGCCAGTTAATAGTTTGCGCAACGTTGTTGCCATTGCTACAGGCATCGT
GGTGTCACGCTCGTCGTTTGGTATGGCTTCATTCAGCTCCGGTTCCCAACGATCAA
GGCGAGTTACATGATCCCCCATGTTGTGCAAAAAAGCGGTTAGCTCCTTCGGTCCT
CCGATCGTTGTCAGAAGTAAGTTGGCCGCAGTGTTATCACTCATGGTTATGGCAGC
ACTGCATAATTCTCTTACTGTCATGCCATCCGTAAGATGCTTTTCTGTGACTGGTGA
GTACTCAACCAAGTCATTCTGAGAATAGTGTATGCGGCGACCGAGTTGCTCTTGCC
CGGCGTCAATACGGGATAATACCGCGCCACATAGCAGAACTTTAAAAGTGCTCATC
ATTGGAAAACGTTCTTCGGGGCGAAAACTCTCAAGGATCTTACCGCTGTTGAGATC
CAGTTCGATGTAACCCACTCGTGCACCCAACTGATCTTCAGCATCTTTTACTTTCAC
CAGCGTTTCTGGGTGAGCAAAAACAGGAAGGCAAAATGCCGCAAAAAAGGGAATA

AGGGCGACACGGAAATGTTGAATACTCATACTCTTCCTTTTTCAATATTATTGAAGC
ATTTATCAGGGTTATTGTCTCATGAGCGGATACATATTTGAATGTATTTAGAAAAATA
AACAAATAGGGGTTCCGCGCACATTTCCCCGAAAAGTGCCACCTAAATTGTAAGCG
TTAATATTTTGTTAAAATTCGCGTTAAATTTTTGTTAAATCAGCTCATTTTTTAACCAA
TAGGCCGAAATCGGCAAAATCCCTTATAAATCAAAAGAATAGACCGAGATAGGGTT
GAGTGTTGTTCCAGTTTGGAACAAGAGTCCACTATTAAAGAACGTGGACTCCAACG
TCAAAGGGCGAAAAACCGTCTATCAGGGCGATGGCCCACTACGTGAACCATCACC
CTAATCAAGTTTTTTGGGGTCGAGGTGCCGTAAAGCACTAAATCGGAACCCTAAAG
GGAGCCCCCGATTTAGAGCTTGACGGGGAAAGCCGGCGAACGTGGCGAGAAAGG
AAGGGAAGAAAGCGAAAGGAGCGGGCGCTAGGGCGCTGGCAAGTGTAGCGGTCA
CGCTGCGCGTAACCACCACACCCGCCGCGCTTAATGCGCCGCTACAGGGCGCGT
CCCATTCGCCATTCAGGCTGCGCAACTGTTGGGAAGGGCGATCGGTGCGGGCCT
CTTCGCTATTACGCCAGCTGGCGAAAGGGGGATGTGCTGCAAGGCGATTAAGTTG
GGTAACGCCAGGGTTTTCCCAGTCACGACGTTGTAAAACGACGGCCAGTGAATTG
