# Supplementary material for: Leveraging Synthetic Virology for the Rapid Engineering of Vesicular Stomatitis Virus (VSV)
Source: Viruses. 2024 Oct 21;16(10):1641. doi: 10.3390/v16101641 (PMC11512388; doi:10.3390/v16101641)
Supplement: Supplementary file 1 [file viruses-16-01641-s001.zip › Supplementary Table S1.pdf]

Fragment #	Length (bp)	Sequence
F1	4,450	GGGTCGGCATGGCATCTCCACCTCCTCGCGGTCCGACCTGGGCATCCGAAGGAGGA<br>CGTCGTCCACTCGGATGGCTAAGGGAGGGGCCCCCGCGGGGCTGCTAACAAAGCCC<br>GAAAGGAAGCTGAGTTGGCTGCTGCCACCGCTGAGCAATAACTAGCATAACCCCTTG<br>GGGCCTCTAAACGGGTCTTGAGGGGTTTTTTGCTGAAAGGAGGAACTATATCCGGATC<br>GAGACCTCGATACTAGTGCGGTGGAGCTCCAGCTTTTGTTCCCTTTAGTGAGGGTTAA<br>TTTCGAGCTTGGCGTAATCATGGTCATAGCTGTTTCCTGTGTGAAATTGTTATCCGCTC<br>ACAATTCCACACAACATACGAGCCGGAAGCATAAAGTGTAAAGCCTGGGGTGCCTAAT<br>GAGTGAGCTAACTCACATTAATTGCGTTGCGCTCACTGCCCGCTTTCCAGTCGGGAAA<br>CCTGTCGTGCCAGCTGCATTAATGAATCGGCCAACGCGCGGGGAGAGGCGGTTTGC<br>GTATTGGGCGCTCTTCCGCTTCCTCGCTCACTGACTCGCTGCGCTCGGTCGTTCGGC<br>TGCGGCGAGCGGTATCAGCTCACTCAAAGGCGGTAATACGGTTATCCACAGAATCAGG<br>GGATAACGCAGGAAAGAACATGTGAGCAAAAGGCCAGCAAAAGGCCAGGAACCGTAA<br>AAAGGCCGCGTTGCTGGCGTTTTTCCATAGGCTCCGCCCCCCTGACGAGCATCACAA<br>AAATCGACGCTCAAGTCAGAGGTGGCGAAACCCGACAGGACTATAAAGATACCAGGC<br>GTTTCCCCCTGGAAGCTCCCTCGTGCGCTCTCCTGTTCCGACCCTGCCGCTTACCGG<br>ATACCTGTCCGCCTTTCTCCCTTCGGGAAGCGTGGCGCTTTCTCATAGCTCACGCTGT<br>AGGTATCTCAGTTCGGTGTAGGTCGTTCGCTCCAAGCTGGGCTGTGTGCACGAACCC<br>CCCGTTCAGCCCGACCGCTGCGCCTTATCCGGTAACTATCGTCTTGAGTCCAACCCG<br>GTAAGACACGACTTATCGCCACTGGCAGCAGCCACTGGTAACAGGATTAGCAGAGCG<br>AGGTATGTAGGCGGTGCTACAGAGTTCTTGAAGTGGTGGCCTAACTACGGCTACACTA<br>GAAGAACAGTATTTGGTATCTGCGCTCTGCTGAAGCCAGTTACCTTCGGAAAAAGAGT<br>TGGTAGCTCTTGATCCGGCAAACAAACCACCGCTGGTAGCGGTGGTTTTTTTGTTTGC<br>AAGCAGCAGATTACGCGCAGAAAAAAAGGATCTCAAGAAGATCCTTTGATCTTTTCTAC<br>GGGGTCTGACGCTCAGTGGAACGAAAACTCACGTTAAGGGATTTTGGTCATGAGATTA<br>TCAAAAAGGATCTTCACCTAGATCCTTTTAAATTAAAAATGAAGTTTTAAATCAATCTAAA<br>GTATATATGAGTAAACTTGGTCTGACAGTTACCAATGCTTAATCAGTGAGGCACCTATCT<br>CAGCGATCTGTCTATTTCGTTCATCCATAGTTGCCTGACTCCCCGTCGTGTAGATAACT<br>ACGATACGGGAGGGCTTACCATCTGGCCCCAGTGCTGCAATGATACCGCGAGACCCA<br>CGCTCACCGGCTCCAGATTTATCAGCAATAAACCAGCCAGCCGGAAGGGCCGAGCGC<br>AGAAGTGGTCCTGCAACTTTATCCGCCTCCATCCAGTCTATTAATTGTTGCCGGGAAGC<br>TAGAGTAAGTAGTTCGCCAGTTAATAGTTTGCGCAACGTTGTTGCCATTGCTACAGGCA<br>TCGTGGTGTCACGCTCGTCGTTTGGTATGGCTTCATTCAGCTCCGGTTCCCAACGATC<br>AAGGCGAGTTACATGATCCCCCATGTTGTGCAAAAAAGCGGTTAGCTCCTTCGGTCCT<br>CCGATCGTTGTCAGAAGTAAGTTGGCCGCAGTGTTATCACTCATGGTTATGGCAGCAC<br>TGCATAATTCTCTTACTGTCATGCCATCCGTAAGATGCTTTTCTGTGACTGGTGAGTACT<br>CAACCAAGTCATTCTGAGAATAGTGTATGCGGCGACCGAGTTGCTCTTGCCCGGCGTC<br>AATACGGGATAATACCGCGCCACATAGCAGAACTTTAAAAGTGCTCATCATTGGAAAAC<br>GTTCTTCGGGGCGAAAACTCTCAAGGATCTTACCGCTGTTGAGATCCAGTTCGATGTA<br>ACCCACTCGTGCACCCAACTGATCTTCAGCATCTTTTACTTTCACCAGCGTTTCTGGGT<br>GAGCAAAAACAGGAAGGCAAAATGCCGCAAAAAAGGGAATAAGGGCGACACGGAAAT<br>GTTGAATACTCATACTCTTCCTTTTTCAATATTATTGAAGCATTTATCAGGGTTATTGTCTC<br>ATGAGCGGATACATATTTGAATGTATTTAGAAAAATAAACAAATAGGGGTTCCGCGCACA<br>TTTCCCCGAAAAGTGCCACCTAAATTGTAAGCGTTAATATTTTGTTAAAATTCGCGTTAA<br>ATTTTTGTTAAATCAGCTCATTTTTTAACCAATAGGCCGAAATCGGCAAAATCCCTTATAA<br>ATCAAAAGAATAGACCGAGATAGGGTTGAGTGTTGTTCCAGTTTGGAACAAGAGTCCA<br>CTATTAAAGAACGTGGACTCCAACGTCAAAGGGCGAAAAACCGTCTATCAGGGCGATG<br>GCCCACTACGTGAACCATCACCCTAATCAAGTTTTTTGGGGTCGAGGTGCCGTAAAGC<br>ACTAAATCGGAACCCTAAAGGGAGCCCCCGATTTAGAGCTTGACGGGGAAAGCCGGC<br>GAACGTGGCGAGAAAGGAAGGGAAGAAAGCGAAAGGAGCGGGCGCTAGGGCGCTG<br>GCAAGTGTAGCGGTCACGCTGCGCGTAACCACCACACCCGCCGCGCTTAATGCGCC<br>GCTACAGGGCGCGTCCCATTCGCCATTCAGGCTGCGCAACTGTTGGGAAGGGCGATC<br>GGTGCGGGCCTCTTCGCTATTACGCCAGCTGGCGAAAGGGGGATGTGCTGCAAGGC<br>GATTAAGTTGGGTAACGCCAGGGTTTTCCCAGTCACGACGTTGTAAAACGACGGCCA<br>GTGAATTGTAATACGACTCACTATAGGACGAAGACAAACAAACCATTATTATCATTAAAA<br>GGCTCAGGAGAAACTTTAACAGTAATCAAAATGTCTGTTACAGTCAAGAGAATCATTGA<br>CAACACAGTCGTAGTTCCAAAACTTCCTGCAAATGAGGATCCAGTGGAATACCCGGCA<br>GATTACTTCAGAAAATCAAAGGAGATTCCTCTTTACATCAATACTACAAAAAGTTTGTCA<br>GATCTAAGAGGATATGTCTACCAAGGCCTCAAATCCGGAAATGTATCAATCATACATGTC<br>AACAGCTACTTGTATGGAGCATTAAAGGACATCCGGGGTAAGTTGGATAAAGATTGGTC<br>AAGTTTCGGAATAAACATCGGGAAAGCAGGGGATACAATCGGAATATTTGACCTTGTAT<br>CCTTGAAAGCCCTGGACGGCGTACTTCCAGATGGAGTATCGGATGCTTCCAGAACCA<br>GCGCAGATGACAAATGGTTGCCTTTGTATCTACTTGGCTTATACAGAGTGGGCAGAACA<br>CAAATGCCTGAATACAGAAAAAAGCTCATGGATGGGCTGACAAATCAATGCAAAATGAT<br>CAATGAACAGTTTGAACCTCTTGTGCCAGAAGGTCGTGACATTTTTGATGTGTGGGGA<br>AATGACAGTAATTACACAAAAATTGTCGCTGCAGTGGACATGTTCTTCCACATGTTCAA<br>AAAACATGAATGTGCCTCGTTCAGATACGGAACTATTGTTTCCAGATTCAAAGATTGTG<br>CTGCATTGGCAACATTTGGACACCTCTGCAAAATAACCGGAATGTCTACAGAAGATGTA<br>ACGACCTGGATCTTGAACCGAGAAGTTGCAGATGAAATGGTCCAAATGATGCTTCCAG<br>GCCAAGAAATTGACAAGGCCGATTCATACATGCCTTATTTGATCGACTTTGGATTGTCTT

		CTAAGTCTCCATATTCTTCCGTCAAAAACCCTGCCTTCCACTTCTGGGGGCAATTGACA<br>GCTCTTCTGCTCAGATCCACCAGAGCAAGGAATGCCCGACAGCCTGATGACATTGAGT<br>ATACATCTCTTACTACAGCAGGTTTGTTGTACGCTTATGCAGTAGGATCCTCTGCCGAC<br>TTGGCACAACAGTTTTGTGTTGGAGATAACAAATACACTCCAGATGATAGTACCGGAGG<br>ATTGACGACTAATGCACCGCCACAAGGCAGAGATGTGGTCGAATGGCTCGGATGGTTT<br>GAAGATCAAAACAGAAAACCGACTCCTGATATGATGCAGTATGCGAAAAGAGCAGTCA<br>TGTCACTGCAAGGCCTAAGAGAGAAGACAATTGGCAAGTATGCTAAGTCAGAATTTGA<br>CAAATGACCCTATAATTCTCAGATCAC
F2	1,751	TGACAAATGACCCTATAATTCTCAGATCACCTATTATATATTATGCTACATATGAAAAAAAC<br>TAACAGATATCATGGATAATCTCACAAAAGTTCGTGAGTATCTCAAGTCCTATTCTCGTC<br>TGGATCAGGCGGTAGGAGAGATAGATGAGATCGAAGCACAACGAGCTGAAAAGTCCA<br>ATTATGAGTTGTTCCAAGAGGATGGAGTGGAAGAGCATACTAAGCCCTCTTATTTTCAG<br>GCAGCAGATGATTCTGACACAGAATCTGAACCAGAAATTGAAGACAATCAAGGCTTGT<br>ATGCACCAGATCCAGAAGCTGAGCAAGTTGAAGGCTTTATACAGGGGCCTTTAGATGA<br>CTATGCAGATGAGGAAGTGGATGTTGTATTTACTTCGGACTGGAAACAGCCTGAGCTT<br>GAATCTGACGAGCATGGAAAGACCTTACGGTTGACATCGCCAGAGGGTTTAAGTGGA<br>GAGCAGAAATCCCAGTGGCTTTCGACGATTAAAGCAGTCGTGCAAAGTGCCAAATACT<br>GGAATCTGGCAGAGTGCACATTTGAAGCATCGGGAGAAGGGGTCATTATGAAGGAGC<br>GCCAGATAACTCCGGATGTATATAAGGTCACTCCAGTGATGAACACACATCCGTCCCAA<br>TCAGAAGCAGTATCAGATGTTTGGTCTCTCTCAAAGACATCCATGACTTTCCAACCCAA<br>GAAAGCAAGTCTTCAGCCTCTCACCATATCCTTGGATGAATTGTTCTCATCTAGAGGAG<br>AGTTCATCTCTGTCGGAGGTGACGGACGAATGTCTCATAAAGAGGCCATCCTGCTCGG<br>CCTGAGATACAAAAAGTTGTACAATCAGGCGAGAGTCAAATATTCTCTGTAGACTATGA<br>AAAAAAGTAACAGATATCACGATCTAAGTGTTATCCCAATCCATTCATCATGAGTTCCTTA<br>AAGAAGATTCTCGGTCTGAAGGGGAAAGGTAAGAAATCTAAGAAATTAGGGATCGCAC<br>CACCCCCTTATGAAGAGGACACTAGCATGGAGTATGCTCCGAGCGCTCCAATTGACAA<br>ATCCTATTTTGGAGTTGACGAGATGGACACCTATGATCCGAATCAATTAAGATATGAGAA<br>ATTCTTCTTTACAGTGAAAATGACGGTTAGATCTAATCGTCCGTTCAGAACATACTCAGA<br>TGTGGCAGCCGCTGTATCCCATTGGGATCACATGTACATCGGAATGGCAGGGAAACGT<br>CCCTTCTACAAAATCTTGGCTTTTTTGGGTTCTTCTAATCTAAAGGCCACTCCAGCGGT<br>ATTGGCAGATCAAGGTCAACCAGAGTATCACGCTCACTGCGAAGGCAGGGCTTATTTG<br>CCACATAGGATGGGGAAGACCCCTCCCATGCTCAATGTACCAGAGCACTTCAGAAGAC<br>CATTCAATATAGGTCTTTACAAGGGAACGATTGAGCTCACAATGACCATCTACGATGATG<br>AGTCACTGGAAGCAGCTCCTATGATCTGGGATCATTTCAATTCTTCCAAATTTTCTGATT<br>TCAGAGAGAAGGCCTTAATGTTTGGCCTGATTGTCGAGAAAAAGGCATCTGGAGCGTG<br>GGTCCTGGACTCTATCGGCCACTTCAAATGAGCTAGTCTAACTTCTAGCTTCTGAACAA<br>TCCCCGGTTTACTCAGTCTCCCCTAATTCCAGCCTCTCGAACAACTAATATCCTGTCTT<br>TTCTATCCCTATGAAAAAAACTAACAGAGATCGATCTGTTTACGCGT
F3	1,709	AAACTAACAGAGATCGATCTGTTTACGCGTCACTATGAAGTGCCTTTTGTACTTAGCCTT<br>TTTATTCATTGGGGTGAATTGCAAGTTCACCATAGTTTTTCCACACAACCAAAAAGGAA<br>ACTGGAAAAATGTTCCTTCTAATTACCATTATTGCCCGTCAAGCTCAGATTTAAATTGGC<br>ATAATGACTTAATAGGCACAGCCTTACAAGTCAAAATGCCCAAGAGTCACAAGGCTATT<br>CAAGCAGACGGTTGGATGTGTCATGCTTCCAAATGGGTCACTACTTGTGATTTCCGCT<br>GGTATGGACCGAAGTATATAACACATTCCATCCGATCCTTCACTCCATCTGTAGAACAAT<br>GCAAGGAAAGCATTGAACAAACGAAACAAGGAACTTGGCTGAATCCAGGCTTCCCTC<br>CTCAAAGTTGTGGATATGCAACTGTGACGGATGCCGAAGCAGTGATTGTCCAGGTGAC<br>TCCTCACCATGTGCTGGTTGATGAATACACAGGAGAATGGGTTGATTCACAGTTCATCA<br>ACGGAAAATGCAGCAATTACATATGCCCCACTGTCCATAACTCTACAACCTGGCATTCT<br>GACTATAAGGTCAAAGGGCTATGTGATTCTAACCTCATTTCCATGGACATCACCTTCTTC<br>TCAGAGGACGGAGAGCTATCATCCCTGGGAAAGGAGGGCACAGGGTTCAGAAGTAAC<br>TACTTTGCTTATGAAACTGGAGGCAAGGCCTGCAAAATGCAATACTGCAAGCATTGGG<br>GAGTCAGACTCCCATCAGGTGTCTGGTTCGAGATGGCTGATAAGGATCTCTTTGCTGC<br>AGCCAGATTCCCTGAATGCCCAGAAGGGTCAAGTATCTCTGCTCCATCTCAGACCTCA<br>GTGGATGTAAGTCTAATTCAGGACGTTGAGAGGATCTTGGATTATTCCCTCTGCCAAGA<br>AACCTGGAGCAAAATCAGAGCGGGTCTTCCAATCTCTCCAGTGGATCTCAGCTATCTT<br>GCTCCTAAAAACCCAGGAACCGGTCCTGCTTTCACCATAATCAATGGTACCCTAAAATA<br>CTTTGAGACCAGATACATCAGAGTCGATATTGCTGCTCCAATCCTCTCAAGAATGGTCG<br>GAATGATCAGTGGAACTACCACAGAAAGGGAACTGTGGGATGACTGGGCACCATATGA<br>AGACGTGGAAATTGGACCCAATGGAGTTCTGAGGACCAGTTCAGGATATAAGTTTCCT<br>TTATACATGATTGGACATGGTATGTTGGACTCCGATCTTCATCTTAGCTCAAAGGCTCAG<br>GTGTTCGAACATCCTCACATTCAAGACGCTGCTTCGCAACTTCCTGATGATGAGAGTTT<br>ATTTTTTGGTGATACTGGGCTATCCAAAAATCCAATCGAGCTTGTAGAAGGTTGGTTCA<br>GTAGTTGGAAAAGCTCTATTGCCTCTTTTTTCTTTATCATAGGGTTAATCATTGGACTATT<br>CTTGGTTCTCCGAGTTGGTATCCATCTTTGCATTAAATTAAAGCACACCAAGAAAAGAC<br>AGATTTATACAGACATAGAGATGAACCGACTTGGAAAGTAACTCAAATCCTGCTAGCCA<br>GATTCTTCATGTTTGGACCAAATCAACTTGTGATACCATGCTCAAAGAGGCCTCAATTAT<br>ATTTGAGTTTTTAATTTTTATGAAAAAAACTAACAGCAATCATGGAAGTCCACGATTTTGA
F4	6,439	AACAGCAATCATGGAAGTCCACGATTTTGAGACCGACGAGTTCAATGATTTCAATGAAG<br>ATGACTATGCCACAAGAGAATTCCTGAATCCCGATGAGCGCATGACGTACTTGAATCAT<br>GCTGATTACAACCTGAATTCTCCTCTAATTAGTGATGATATTGACAATTTAATCAGGAAAT

		TCAATTCTCTTCCAATTCCCTCGATGTGGGATAGTAAGAACTGGGATGGAGTTCTTGAG<br>ATGTTAACATCATGTCAAGCCAATCCCATCTCAACATCTCAGATGCATAAATGGATGGGA<br>AGTTGGTTAATGTCTGATAATCATGATGCCAGTCAAGGGTATAGTTTTTTACATGAAGTG<br>GACAAAGAGGCAGAAATAACATTTGACGTGGTGGAGACCTTCATCCGCGGCTGGGGC<br>AACAAACCAATTGAATACATCAAAAAGGAAAGATGGACTGACTCATTCAAAATTCTCGC<br>TTATTTGTGTCAAAAGTTTTTGGACTTACACAAGTTGACATTAATCTTAAATGCTGTCTCT<br>GAGGTGGAATTGCTCAACTTGGCGAGGACTTTCAAAGGCAAAGTCAGAAGAAGTTCT<br>CATGGAACGAACATATGCAGGATTAGGGTTCCCAGCTTGGGTCCTACTTTTATTTCAGA<br>AGGATGGGCTTACTTCAAGAAACTTGATATTCTAATGGACCGAAACTTTCTGTTAATGGT<br>CAAAGATGTGATTATAGGGAGGATGCAAACGGTGCTATCCATGGTATGTAGAATAGACA<br>ACCTGTTCTCAGAGCAAGACATCTTCTCCCTTCTAAATATCTACAGAATTGGAGATAAAA<br>TTGTGGAGAGGCAGGGAAATTTTTCTTATGACTTGATTAAAATGGTGGAACCGATATGC<br>AACTTGAAGCTGATGAAATTAGCAAGAGAATCAAGGCCTTTAGTCCCACAATTCCCTCA<br>TTTTGAAAATCATATCAAGACTTCTGTTGATGAAGGGGCAAAAATTGACCGAGGTATAA<br>GATTCCTCCATGATCAGATAATGAGTGTGAAAACAGTGGATCTCACACTGGTGATTTAT<br>GGATCGTTCAGACATTGGGGTCATCCTTTTATAGATTATTACACTGGACTAGAAAAATTA<br>CATTCCCAAGTAACCATGAAGAAAGATATTGATGTGTCATATGCAAAAGCACTTGCAAG<br>TGATTTAGCTCGGATTGTTCTATTTCAACAGTTCAATGATCATAAAAAGTGGTTCGTGAA<br>TGGAGACTTGCTCCCTCATGATCATCCCTTTAAAAGTCATGTTAAAGAAAATACATGGC<br>CCACAGCTGCTCAAGTTCAAGATTTTGGAGATAAATGGCATGAACTTCCGCTGATTAAA<br>TGTTTTGAAATACCCGACTTACTAGACCCATCGATAATATACTCTGACAAAAGTCATTCA<br>ATGAATAGGTCAGAGGTGTTGAAACATGTCCGAATGAATCCGAACACTCCTATCCCTAG<br>TAAAAAGGTGTTGCAGACTATGTTGGACACAAAGGCTACCAATTGGAAAGAATTTCTTA<br>AAGAGATTGATGAGAAGGGCTTAGATGATGATGATCTAATTATTGGTCTTAAAGGAAAG<br>GAGAGGGAACTGAAGTTGGCAGGTAGATTTTTCTCCCTAATGTCTTGGAAATTGCGAG<br>AATACTTTGTAATTACCGAATATTTGATAAAGACTCATTTCGTCCCTATGTTTAAAGGCCT<br>GACAATGGCGGACGATCTAACTGCAGTCATTAAAAAGATGTTAGATTCCTCATCCGGCC<br>AAGGATTGAAGTCATATGAGGCAATTTGCATAGCCAATCACATTGATTACGAAAAATGGA<br>ATAACCACCAAAGGAAGTTATCAAACGGCCCAGTGTTCCGAGTTATGGGCCAGTTCTT<br>AGGTTATCCATCCTTAATCGAGAGAACTCATGAATTTTTTGAGAAAAGTCTTATATACTAC<br>AATGGAAGACCAGACTTGATGCGTGTTCACAACAACACACTGATCAATTCAACCTCCC<br>AACGAGTTTGTTGGCAAGGACAAGAGGGTGGACTGGAAGGTCTACGGCAAAAAGGAT<br>GGAGTATCCTCAATCTACTGGTTATTCAAAGAGAGGCTAAAATCAGAAACACTGCTGTC<br>AAAGTCTTGGCACAAGGTGATAATCAAGTTATTTGCACACAGTATAAAACGAAGAAATC<br>GAGAAACGTTGTAGAATTACAGGGTGCTCTCAATCAAATGGTTTCTAATAATGAGAAAAT<br>TATGACTGCAATCAAAATAGGGACAGGGAAGTTAGGACTTTTGATAAATGACGATGAGA<br>CTATGCAATCTGCAGATTACTTGAATTATGGAAAAATACCGATTTTCCGTGGAGTGATTA<br>GAGGGTTAGAGACCAAGAGATGGTCACGAGTGACTTGTGTCACCAATGACCAAATACC<br>CACTTGTGCTAATATAATGAGCTCAGTTTCCACAAATGCTCTCACCGTAGCTCATTTTGC<br>TGAGAACCCAATCAATGCCATGATACAGTACAATTATTTTGGGACATTTGCTAGACTCTT<br>GTTGATGATGCATGATCCTGCTCTTCGTCAATCATTGTATGAAGTTCAAGATAAGATACC<br>GGGCTTGCACAGTTCTACTTTCAAATACGCCATGTTGTATTTGGACCCTTCCATTGGAG<br>GAGTGTCGGGCATGTCTTTGTCCAGGTTTTTGATTAGAGCCTTCCCAGATCCCGTAAC<br>AGAAAGTCTCTCATTCTGGAGATTCATCCATGTACATGCTCGAAGTGAGCATCTGAAGG<br>AGATGAGTGCAGTATTTGGAAACCCCGAGATAGCCAAGTTTCGAATAACTCACATAGAC<br>AAGCTAGTAGAAGATCCAACCTCTCTGAACATCGCTATGGGAATGAGTCCAGCGAACT<br>TGTTAAAGACTGAGGTTAAAAAATGCTTAATCGAATCAAGACAAACCATCAGGAACCAG<br>GTGATTAAGGATGCAACCATATATTTGTATCATGAAGAGGATCGGCTCAGAAGTTTCTTA<br>TGGTCAATAAATCCTCTGTTCCCTAGATTTTTAAGTGAATTCAAATCAGGCACTTTTTTG<br>GGAGTCGCAGACGGGCTCATCAGTCTATTTCAAAATTCTCGTACTATTCGGAACTCCTT<br>TAAGAAAAAGTATCATAGGGAATTGGATGATTTGATTGTGAGGAGTGAGGTATCCTCTTT<br>GACACATTTAGGGAAACTTCATTTGAGAAGGGGATCATGTAAAATGTGGACATGTTCAG<br>CTACTCATGCTGACACATTAAGATACAAATCCTGGGGCCGTACAGTTATTGGGACAACT<br>GTACCCCATCCATTAGAAATGTTGGGTCCACAACATCGAAAAGAGACTCCTTGTGCAC<br>CATGTAACACATCAGGGTTCAATTATGTTTCTGTGCATTGTCCAGACGGGATCCATGAC<br>GTCTTTAGTTCACGGGGACCATTGCCTGCTTATCTAGGGTCTAAAACATCTGAATCTAC<br>ATCTATTTTGCAGCCTTGGGAAAGGGAAAGCAAAGTCCCACTGATTAAAAGAGCTACA<br>CGTCTTAGAGATGCTATCTCTTGGTTTGTTGAACCCGACTCTAAACTAGCAATGACTATA<br>CTTTCTAACATCCACTCTTTAACAGGCGAAGAATGGACCAAAAGGCAGCATGGGTTCA<br>AAAGAACAGGGTCTGCCCTTCATAGGTTTTCGACATCTCGGATGAGCCATGGTGGGTT<br>CGCATCTCAGAGCACTGCAGCATTGACCAGGTTGATGGCAACTACAGACACCATGAG<br>GGATCTGGGAGATCAGAATTTCGACTTTTTATTCCAAGCAACGTTGCTCTATGCTCAAA<br>TTACCACCACTGTTGCAAGAGACGGATGGATCACCAGTTGTACAGATCATTATCATATT<br>GCCTGTAAGTCCTGTTTGAGACCCATAGAAGAGATCACCCTGGACTCAAGTATGGACT<br>ACACGCCCCCAGATGTATCCCATGTGCTGAAGACATGGAGGAATGGGGAAGGTTCGT<br>GGGGACAAGAGATAAAACAGATCTATCCTTTAGAAGGGAATTGGAAGAATTTAGCACCT<br>GCTGAGCAATCCTATCAAGTCGGCAGATGTATAGGTTTTCTATATGGAGACTTGGCGTA<br>TAGAAAATCTACTCATGCCGAGGACAGTTCTCTATTTCCTCTATCTATACAAGGTCGTAT<br>TAGAGGTCGAGGTTTCTTAAAAGGGTTGCTAGACGGATTAATGAGAGCAAGTTGCTGC<br>CAAGTAATACACCGGAGAAGTCTGGCTCATTTGAAGAGGCCGGCCAACGCAGTGTAC

		GGAGGTTTGATTTACTTGATTGATAAATTGAGTGTATCACCTCCATTCCTTTCTCTTACTA<br>GATCAGGACCTATTAGAGACGAATTAGAAACGATTCCCCACAAGATCCCAACCTCCTAT<br>CCGACAAGCAACCGTGATATGGGGGTGATTGTCAGAAATTACTTCAAATACCAATGCCG<br>TCTAATTGAAAAGGGAAAATACAGATCACATTATTCACAATTATGGTTATTCTCAGATGTC<br>TTATCCATAGACTTCATTGGACCATTCTCTATTTCCACCACCCTCTTGCAAATCCTATACA<br>AGCCATTTTTATCTGGGAAAGATAAGAATGAGTTGAGAGAGCTGGCAAATCTTTCTTCA<br>TTGCTAAGATCAGGAGAGGGGTGGGAAGACATACATGTGAAATTCTTCACCAAGGACA<br>TATTATTGTGTCCAGAGGAAATCAGACATGCTTGCAAGTTCGGGATTGCTAAGGATAATA<br>ATAAAGACATGAGCTATCCCCCTTGGGGAAGGGAATCCAGAGGGACAATTACAACAAT<br>CCCTGTTTATTATACGACCACCCCTTACCCAAAGATGCTAGAGATGCCTCCAAGAATCC<br>AAAATCCCCTGCTGTCCGGAATCAGGTTGGGCCAATTACCAACTGGCGCTCATTATAAA<br>ATTCGGAGTATATTACATGGAATGGGAATCCATTACAGGGACTTCTTGAGTTGTGGAGA<br>CGGCTCCGGAGGGATGACTGCTGCATTACTACGAGAAAATGTGCATAGCAGAGGAATA<br>TTCAATAGTCTGTTAGAATTATCAGGGTCAGTCATGCGAGGCGCCTCTCCTGAGCCCC<br>CCAGTGCCCTAGAAACTTTAGGAGGAGATAAATCGAGATGTGTAAATGGTGAAACATGT<br>TGGGAATATCCATCTGACTTATGTGACCCAAGGACTTGGGACTATTTCCTCCGACTCAA<br>AGCAGGCTTGGGGCTTCAAATTGATTTAATTGTAATGGATATGGAAGTTCGGGATTCTT<br>CTACTAGCCTGAAAATTGAGACGAATGTTAGAAATTATGTGCACCGGATTTTGGATGAG<br>CAAGGAGTTTTAATCTACAAGACTTATGGAACATATATTTGTGAGAGCGAAAAGAATGCA<br>GTAACAATCCTTGGTCCCATGTTCAAGACGGTCGACTTAGTTCAAACAGAATTTAGTAG<br>TTCTCAAACGTCTGAAGTATATATGGTATGTAAAGGTTTGAAGAAATTAATCGATGAACC<br>CAATCCCGATTGGTCTTCCATCAATGAATCCTGGAAAAACCTGTACGCATTCCAGTCAT<br>CAGAACAGGAATTTGCCAGAGCAAAGAAGGTTAGTACATACTTTACCTTGACAGGTATT<br>CCCTCCCAATTCATTCCTGATCCTTTTGTAAACATTGAGACTATGCTACAAATATTCGGA<br>GTACCCACGGGTGTGTCTCATGCGGCTGCCTTAAAATCATCTGATAGACCTGCAGATTT<br>ATTGACCATTAGCCTTTTTTATATGGCGATTATATCGTATTATAACATCAATCATATCAGAG<br>TAGGACCGATACCTCCGAACCCCCCATCAGATGGAATTGCACAAAATGTGGGGATCGC<br>TATAACTGGTATAAGCTTTTGGCTGAGTTTGATGGAGAAAGACATTCCACTATATCAACA<br>GTGTTTAGCAGTTATCCAGCAATCATTCCCGATTAGGTGGGAGGCTGTTTCAGTAAAAG<br>GAGGATACAAGCAGAAGTGGAGTACTAGAGGTGATGGGCTCCCAAAAGATACCCGAAT<br>TTCAGACTCCTTGGCCCCAATCGGGAACTGGATCAGATCTCTGGAATTGGTCCGAAAC<br>CAAGTTCGTCTAAATCCATTCAATGAGATCTTGTTCAATCAGCTATGTCGTACAGTGGAT<br>AATCATTTGAAATGGTCAAATTTGCGAAGAAACACAGGAATGATTGAATGGATCAATAGA<br>CGAATTTCAAAAGAAGACCGGTCTATACTGATGTTGAAGAGTGACCTACACGAGGAAA<br>ACTCTTGGAGAGATTAAAAAATCATGAGGAGACTCCAAACTTTAAGTATGAAAAAAACTT<br>TGATCCTTAAGACCCTCTTGTGGTTTTTATTTTTTATCTGGTTTTGTGGTCTTCGT
