# Supplementary material for: Leveraging Synthetic Virology for the Rapid Engineering of Vesicular Stomatitis Virus (VSV)
Source: Viruses. 2024 Oct 21;16(10):1641. doi: 10.3390/v16101641 (PMC11512388; doi:10.3390/v16101641)
Supplement: Supplementary file 1 [file viruses-16-01641-s001.zip › Supplementary Table S2.pdf]

Fragment #	Length (bp)	Sequence
F1	4,450	gggtcggcatggcatctccacctcctcgcggtccgacctgggcatccgaaggaggacgtcgtccactcggatggctaaggg<br>aggggcccccgcggggctgctaacaaagcccgaaaggaagctgagttggctgctgccaccgctgagcaataactagcata<br>accccttggggcctctaaacgggtcttgaggggttttttgctgaaaggaggaactatatccggatcgagacctcgatactagtgc<br>ggtggagctccagcttttgttccctttagtgagggttaatttcgagcttggcgtaatcatggtcatagctgtttcctgtgtgaaattgtta<br>tccgctcacaattccacacaacatacgagccggaagcataaagtgtaaagcctggggtgcctaatgagtgagctaactcaca<br>ttaattgcgttgcgctcactgcccgctttccagtcgggaaacctgtcgtgccagctgcattaatgaatcggccaacgcgcgggg<br>agaggcggtttgcgtattgggcgctcttccgcttcctcgctcactgactcgctgcgctcggtcgttcggctgcggcgagcggtatc<br>agctcactcaaaggcggtaatacggttatccacagaatcaggggataacgcaggaaagaacatgtgagcaaaaggccag<br>caaaaggccaggaaccgtaaaaaggccgcgttgctggcgtttttccataggctccgcccccctgacgagcatcacaaaaat<br>cgacgctcaagtcagaggtggcgaaacccgacaggactataaagataccaggcgtttccccctggaagctccctcgtgcgc<br>tctcctgttccgaccctgccgcttaccggatacctgtccgcctttctcccttcgggaagcgtggcgctttctcatagctcacgctgta<br>ggtatctcagttcggtgtaggtcgttcgctccaagctgggctgtgtgcacgaaccccccgttcagcccgaccgctgcgccttatc<br>cggtaactatcgtcttgagtccaacccggtaagacacgacttatcgccactggcagcagccactggtaacaggattagcaga<br>gcgaggtatgtaggcggtgctacagagttcttgaagtggtggcctaactacggctacactagaagaacagtatttggtatctgc<br>gctctgctgaagccagttaccttcggaaaaagagttggtagctcttgatccggcaaacaaaccaccgctggtagcggtggttttt<br>ttgtttgcaagcagcagattacgcgcagaaaaaaaggatctcaagaagatcctttgatcttttctacggggtctgacgctcagtg<br>gaacgaaaactcacgttaagggattttggtcatgagattatcaaaaaggatcttcacctagatccttttaaattaaaaatgaagttt<br>taaatcaatctaaagtatatatgagtaaacttggtctgacagttaccaatgcttaatcagtgaggcacctatctcagcgatctgtct<br>atttcgttcatccatagttgcctgactccccgtcgtgtagataactacgatacgggagggcttaccatctggccccagtgctgcaa<br>tgataccgcgagacccacgctcaccggctccagatttatcagcaataaaccagccagccggaagggccgagcgcagaag<br>tggtcctgcaactttatccgcctccatccagtctattaattgttgccgggaagctagagtaagtagttcgccagttaatagtttgcgc<br>aacgttgttgccattgctacaggcatcgtggtgtcacgctcgtcgtttggtatggcttcattcagctccggttcccaacgatcaagg<br>cgagttacatgatcccccatgttgtgcaaaaaagcggttagctccttcggtcctccgatcgttgtcagaagtaagttggccgcagt<br>gttatcactcatggttatggcagcactgcataattctcttactgtcatgccatccgtaagatgcttttctgtgactggtgagtactcaa<br>ccaagtcattctgagaatagtgtatgcggcgaccgagttgctcttgcccggcgtcaatacgggataataccgcgccacatagc<br>agaactttaaaagtgctcatcattggaaaacgttcttcggggcgaaaactctcaaggatcttaccgctgttgagatccagttcgat<br>gtaacccactcgtgcacccaactgatcttcagcatcttttactttcaccagcgtttctgggtgagcaaaaacaggaaggcaaaat<br>gccgcaaaaaagggaataagggcgacacggaaatgttgaatactcatactcttcctttttcaatattattgaagcatttatcagg<br>gttattgtctcatgagcggatacatatttgaatgtatttagaaaaataaacaaataggggttccgcgcacatttccccgaaaagtg<br>ccacctaaattgtaagcgttaatattttgttaaaattcgcgttaaatttttgttaaatcagctcattttttaaccaataggccgaaatcg<br>gcaaaatcccttataaatcaaaagaatagaccgagatagggttgagtgttgttccagtttggaacaagagtccactattaaaga<br>acgtggactccaacgtcaaagggcgaaaaaccgtctatcagggcgatggcccactacgtgaaccatcaccctaatcaagttt<br>tttggggtcgaggtgccgtaaagcactaaatcggaaccctaaagggagcccccgatttagagcttgacggggaaagccggc<br>gaacgtggcgagaaaggaagggaagaaagcgaaaggagcgggcgctagggcgctggcaagtgtagcggtcacgctgc<br>gcgtaaccaccacacccgccgcgcttaatgcgccgctacagggcgcgtcccattcgccattcaggctgcgcaactgttggga<br>agggcgatcggtgcgggcctcttcgctattacgccagctggcgaaagggggatgtgctgcaaggcgattaagttgggtaacg<br>ccagggttttcccagtcacgacgttgtaaaacgacggccagtgaattgtaatacgactcactataggacgaagacaaacaaa<br>ccattattatcattaaaggctcaggagaaactttaacagtaatcaaaatgtctgttacagtcaagagaatcattgacaacaca<br>gtcgtagttccaaaacttcctgcaaatgaggatccagtggaatacccggcagattacttcagaaaatcaaaggagattcctcttt<br>acatcaatactacaaaaagtttgtcagatctaagaggatatgtctaccaaggcctcaaatccggaaatgtatcaatcatacatgt<br>caacagctacttgtatggagcattaaaggacatccggggtaagttggataaagattggtcaagtttcggaataaacatcggga<br>aagcaggggatacaatcggaatatttgaccttgtatccttgaaagccctggacggcgtacttccagatggagtatcggatgcttc<br>cagaaccagcgcagatgacaaatggttgcctttgtatctacttggcttatacagagtgggcagaacacaaatgcctgaataca<br>gaaaaaagctcatggatgggctgacaaatcaatgcaaaatgatcaatgaacagtttgaacctcttgtgccagaaggtcgtga<br>catttttgatgtgtggggaaatgacagtaattacacaaaaattgtcgctgcagtggacatgttcttccacatgttcaaaaaacatg<br>aatgtgcctcgttcagatacggaactattgtttccagattcaaagattgtgctgcattggcaacatttggacacctctgcaaaataa<br>ccggaatgtctacagaagatgtaacgacctggatcttgaaccgagaagttgcagatgaaatggtccaaatgatgcttccaggc<br>caagaaattgacaaggccgattcatacatgccttatttgatcgactttggattgtcttctaagtctccatattcttccgtcaaaaaccc<br>tgccttccacttctgggggcaattgacagctcttctgctcagatccaccagagcaaggaatgcccgacagcctgatgacattga<br>gtatacatctcttactacagcaggtttgttgtacgcttatgcagtaggatcctctgccgacttggcacaacagttttgtgttggagata<br>acaaatacactccagatgatagtaccggaggattgacgactaatgcaccgccacaaggcagagatgtggtcgaatggctcg<br>gatggtttgaagatcaaaacagaaaaccgactcctgatatgatgcagtatgcgaaaagagcagtcatgtcactgcaaggcct<br>aagagagaagacaattggcaagtatgctaagtcagaatttgacaaatgaccctataattctcagatcac
F2	1,751	tgacaaatgaccctataattctcagatcacctattatatattatgctacatatgaaaaaaactaacagatatcatggataatctcac<br>aaaagttcgtgagtatctcaagtcctattctcgtctggatcaggcggtaggagagatagatgagatcgaagcacaacgagctg<br>aaaagtccaattatgagttgttccaagaggatggagtggaagagcatactaagccctcttattttcaggcagcagatgattctga<br>cacagaatctgaaccagaaattgaagacaatcaaggcttgtatgcaccagatccagaagctgagcaagttgaaggctttata<br>caggggcctttagatgactatgcagatgaggaagtggatgttgtatttacttcggactggaaacagcctgagcttgaatctgacg<br>agcatggaaagaccttacggttgacatcgccagagggtttaagtggagagcagaaatcccagtggctttcgacgattaaagc<br>agtcgtgcaaagtgccaaatactggaatctggcagagtgcacatttgaagcatcgggagaaggggtcattatgaaggagcg<br>ccagataactccggatgtatataaggtcactccagtgatgaacacacatccgtcccaatcagaagcagtatcagatgtttggtc<br>tctctcaaagacatccatgactttccaacccaagaaagcaagtcttcagcctctcaccatatccttggatgaattgttctcatctag<br>aggagagttcatctctgtcggaggtgacggacgaatgtctcataaagaggccatcctgctcggcctgagatacaaaaagttgt<br>acaatcaggcgagagtcaaatattctctgtagactatgaaaaaaagtaacagatatcacgatctaagtgttatcccaatccattc<br>atcatgagttccttaaagaagattctcggtctgaaggggaaaggtaagaaatctaagaaattagggatcgcaccaccccctta<br>tgaagaggacactagcatggagtatgctccgagcgctccaattgacaaatcctattttggagttgacgagatggacacctatga<br>tccgaatcaattaagatatgagaaattcttctttacagtgaaaatgacggttagatctaatcgtccgttcagaacatactcagatgt<br>ggcagccgctgtatcccattgggatcacatgtacatcggaatggcagggaaacgtcccttctacaaaatcttggctttttgggtt<br>cttctaatctaaaggccactccagcggtattggcagatcaaggtcaaccagagtatcacgctcactgcgaaggcagggcttatt

		tgccacataggatggggaagacccctcccatgctcaatgtaccagagcacttcagaagaccattcaatataggtctttacaag<br>ggaacgattgagctcacaatgaccatctacgatgatgagtcactggaagcagctcctatgatctgggatcatttcaattcttcca<br>aattttctgatttcagagagaaggccttaatgtttggcctgattgtcgagaaaaaggcatctggagcgtgggtcctggactctatc<br>ggccacttcaaatgagctagtctaacttctagcttctgaacaatccccggtttactcagtctcccctaattccagcctctcgaacaa<br>ctaatatcctgtcttttctatccctatgaaaaaaactaacagagatcgatctgtttacgcgt
F3	3,111	aaactaacagagatcgatctgtttacgcgtatggcgtccgcagcaccactggtcacggcaatgtgtttgctcggaaatgtgagc<br>ttcccatgcgaccgcccgcccacatgctatacccgcgaaccttccagagccctcgacatccttgaagagaacgtgaaccatg<br>aggcctacgataccctgctcaatgccatattgcggtgcggatcgtctggcagaagcaaaagaagcgtcatcgacgactttacc<br>ctgaccagcccctacttgggcacatgctcgtactgccaccatactgaaccgtgcttcagccctgttaagatcgagcaggtctgg<br>gacgaagcggacgataacaccatacgcatacagacttccgcccagtttggatacgaccatagcggagcagcaagcgcaa<br>acaagtaccgctacatgtcgcttaagcaggatcacaccgttaaagaaggcaccatggatgacatcaagattagcacctcag<br>gaccgtgtagaaggcttagctacaaaggatactttctcctcgcaaaatgccctccaggggacagcgtaacggttagcatagtg<br>agtagcaactcagcaacgtcatgtacactggcccgcaagataaaaccaaaattcgtgggacgggaaaaatatgatctacct<br>cccgttcacggtaaaaaaattccttgcacagtgtacgaccgtctgaaagaaacaactgcaggctacatcactatgcacaggc<br>cgggaccgcacgcttatacatcctacctggaagaatcatcagggaaagtttacgcaaagccgccatctgggaagaacattac<br>gtatgagtgcaagtgcggcgactacaagaccggaaccgtttcgacccgcaccgaaatcactggttgcaccgccatcaagca<br>gtgcgtcgcctataagagcgaccaaacgaagtgggtcttcaactcaccggacttgatcagacatgacgaccacacggccca<br>agggaaattgcatttgcctttcaagttgatcccgagtacctgcatggtccctgttgcccacgcgccgaatgtaatacatggctttaa<br>acacatcagcctccaattagatacagaccacttgacattgctcaccaccaggagactaggggcaaacccggaaccaacca<br>ctgaatggatcgtcggaaagacggtcagaaacttcaccgtcgaccgagatggcctggaatacatatggggaaatcatgagc<br>cagtgagggtctatgcccaagagtcagcaccaggagaccctcacggatggccacacgaaatagtacagcattactaccatc<br>gccatcctgtgtacaccatcttagccgtcgcatcagctaccgtggcgatgatgattggcgtaactgttgcagtgttatgtgcctgta<br>aagcgcgccgtgagtgcctgacgccatacgccctggccccaaacgccgtaatcccaacttcgctggcactcttgtgctgcgtt<br>aggtcggccaatgctgaaacgttcaccgagaccatgagttacttgtggtcgaacagtcagccgttcttctgggtccagttgtgca<br>tacctttggccgctttcatcgttctaatgcgctgctgctcctgctgcctgcctttttttagtggttgccggcgcctacctggcgaaggtag<br>acgcctacgaacatgcgaccactgttccaaatgtgccacagataccgtataaggcacttgttgaaagggcagggtatgcccc<br>gctcaatttggagatcactgtcatgtcctcggaggttttgccttccaccaaccaagagtacattacctgcaaattcaccactgtggt<br>cccctccccaaaaatcaaatgctgcggctccttggaatgtcagccggccgctcatgcagactatacctgcaaggtcttcggag<br>gggtctacccctttatgtggggaggagcgcaatgtttttgcgacagtgagaacagccagatgagtgaggcgtacgtcgaattgt<br>cagcagattgcgcgtctgaccacgcgcaggcgattaaggtgcacactgccgcgatgaaagtaggactgcgtattgtgtacgg<br>gaacactaccagtttcctagatgtgtacgtgaacggagtcacaccaggaacgtctaaagacttgaaagtcatagctggacca<br>atttcagcatcatttacgccattcgatcataaggtcgttatccatcgcggcctggtgtacaactatgacttcccggaatatggagcg<br>atgaaaccaggagcgtttggagacattcaagctacctccttgactagcaaggatctcatcgccagcacagacattaggctact<br>caagccttccgccaagaatgtgcatgtcccgtacacgcaggccgcatcaggatttgagatgtggaaaaacaactcaggccg<br>cccattgcaggaaaccgcacctttcgggtgtaagattgcagtaaatccgctccgagcggtggactgttcatacgggaacattcc<br>catttctattgacatcccgaacgctgcctttatcaggacatcagatgcaccactggtctcaacagtcaaatgtgaagtcagtgagt<br>gcacttattcagcagacttcgacgggatggccaccctgcagtatgtatccgaccgcgaaggtcaatgccccgtacattcgcatt<br>cgagcacagcaactctccaagagtcgacagtacatgtcctggagaaaggagcggtgacagtacactttagcaccgcgagtc<br>cacaggcgaactttatcgtatcgctgtgtgggaagaagacaacatgcaatgcagaatgtaaaccaccagctgaccatatcgt<br>gagcaccccgcacaaaaatgaccaagaatttcaagccgccatctcaaaaacatcatggagttggctgtttgcccttttcggcg<br>gcgcctcgtcgctattaattataggacttatgatttttgcttgcagcatgatgctgactagcacacgaagatgagctagccagattc<br>ttcatgtttggaccaaatcaacttgtgataccatgctcaaagaggcctcaattatatttgagtttttaatttttatgaaaaaaactaac<br>agcaatcatggaagtccacgattttga
F4	6,439	aacagcaatcatggaagtccacgattttgagaccgacgagttcaatgatttcaatgaagatgactatgccacaagagaattcct<br>gaatcccgatgagcgcatgacgtacttgaatcatgctgattacaacctgaattctcctctaattagtgatgatattgacaatttaatc<br>aggaaattcaattctcttccaattccctcgatgtgggatagtaagaactgggatggagttcttgagatgttaacatcatgtcaagc<br>caatcccatctcaacatctcagatgcataaatggatgggaagttggttaatgtctgataatcatgatgccagtcaagggtatagtt<br>ttttacatgaagtggacaaagaggcagaaataacatttgacgtggtggagaccttcatccgcggctggggcaacaaaccaat<br>tgaatacatcaaaaaggaaagatggactgactcattcaaaattctcgcttatttgtgtcaaaagtttttggacttacacaagttgac<br>attaatcttaaatgctgtctctgaggtggaattgctcaacttggcgaggactttcaaaggcaaagtcagaagaagttctcatgga<br>acgaacatatgcaggattagggttcccagcttgggtcctacttttatttcagaaggatgggcttacttcaagaaacttgatattcta<br>atggaccgaaactttctgttaatggtcaaagatgtgattatagggaggatgcaaacggtgctatccatggtatgtagaatagac<br>aacctgttctcagagcaagacatcttctcccttctaaatatctacagaattggagataaaattgtggagaggcagggaaatttttc<br>ttatgacttgattaaaatggtggaaccgatatgcaacttgaagctgatgaaattagcaagagaatcaaggcctttagtcccaca<br>attccctcattttgaaaatcatatcaagacttctgttgatgaaggggcaaaaattgaccgaggtataagattcctccatgatcaga<br>taatgagtgtgaaaacagtggatctcacactggtgatttatggatcgttcagacattggggtcatccttttatagattattacactgg<br>actagaaaaattacattcccaagtaaccatgaagaaagatattgatgtgtcatatgcaaaagcacttgcaagtgatttagctcg<br>gattgttctatttcaacagttcaatgatcataaaaagtggttcgtgaatggagacttgctccctcatgatcatccctttaaaagtcatg<br>ttaaagaaaatacatggcccacagctgctcaagttcaagattttggagataaatggcatgaacttccgctgattaaatgttttgaa<br>atacccgacttactagacccatcgataatatactctgacaaaagtcattcaatgaataggtcagaggtgttgaaacatgtccga<br>atgaatccgaacactcctatccctagtaaaaaggtgttgcagactatgttggacacaaaggctaccaattggaaagaatttctta<br>aagagattgatgagaagggcttagatgatgatgatctaattattggtcttaaaggaaaggagagggaactgaagttggcaggt<br>agatttttctccctaatgtcttggaaattgcgagaatactttgtaattaccgaatatttgataaagactcatttcgtccctatgtttaaag<br>gcctgacaatggcggacgatctaactgcagtcattaaaaagatgttagattcctcatccggccaaggattgaagtcatatgagg<br>caatttgcatagccaatcacattgattacgaaaaatggaataaccaccaaaggaagttatcaaacggcccagtgttccgagtt<br>atgggccagttcttaggttatccatccttaatcgagagaactcatgaatttttttgagaaaagtcttatatactacaatggaagacca<br>gacttgatgcgtgttcacaacaacacactgatcaattcaacctcccaacgagtttgttggcaaggacaagagggtggactgga<br>aggtctacggcaaaaaggatggagtatcctcaatctactggttattcaaagagaggctaaaatcagaaacactgctgtcaaa<br>gtcttggcacaaggtgataatcaagttatttgcacacagtataaaacgaagaaatcgagaaacgttgtagaattacagggtgct<br>ctcaatcaaatggtttctaataatgagaaaattatgactgcaatcaaaatagggacagggaagttaggacttttgataaatgac

		gatgagactatgcaatctgcagattacttgaattatggaaaaataccgattttccgtggagtgattagagggttagagaccaag<br>agatggtcacgagtgacttgtgtcaccaatgaccaaatacccacttgtgctaatataatgagctcagtttccacaaatgctctcac<br>cgtagctcattttgctgagaacccaatcaatgccatgatacagtacaattattttgggacatttgctagactcttgttgatgatgcatg<br>atcctgctcttcgtcaatcattgtatgaagttcaagataagataccgggcttgcacagttctactttcaaatacgccatgttgtatttg<br>gacccttccattggaggagtgtcgggcatgtctttgtccaggtttttgattagagccttcccagatcccgtaacagaaagtctctcat<br>tctggagattcatccatgtacatgctcgaagtgagcatctgaaggagatgagtgcagtatttggaaaccccgagatagccaag<br>tttcgaataactcacatagacaagctagtagaagatccaacctctctgaacatcgctatgggaatgagtccagcgaacttgtta<br>aagactgaggttaaaaaatgcttaatcgaatcaagacaaaccatcaggaaccaggtgattaaggatgcaaccatatatttgta<br>tcatgaagaggatcggctcagaagtttcttatggtcaataaatcctctgttccctagatttttaagtgaattcaaatcaggcactttttt<br>gggagtcgcagacgggctcatcagtctatttcaaaattctcgtactattcggaactcctttaagaaaaagtatcatagggaattg<br>gatgatttgattgtgaggagtgaggtatcctctttgacacatttagggaaacttcatttgagaaggggatcatgtaaaatgtggac<br>atgttcagctactcatgctgacacattaagatacaaatcctggggccgtacagttattgggacaactgtaccccatccattagaa<br>atgttgggtccacaacatcgaaaagagactccttgtgcaccatgtaacacatcagggttcaattatgtttctgtgcattgtccaga<br>cgggatccatgacgtctttagttcacggggaccattgcctgcttatctagggtctaaaacatctgaatctacatctattttgcagcctt<br>gggaaagggaaagcaaagtcccactgattaaaagagctacacgtcttagagatgctatctcttggtttgttgaacccgactcta<br>aactagcaatgactatactttctaacatccactctttaacaggcgaagaatggaccaaaaggcagcatgggttcaaaagaac<br>agggtctgcccttcataggttttcgacatctcggatgagccatggtgggttcgcatctcagagcactgcagcattgaccaggttg<br>atggcaactacagacaccatgagggatctgggagatcagaatttcgactttttattccaagcaacgttgctctatgctcaaattac<br>caccactgttgcaagagacggatggatcaccagttgtacagatcattatcatattgcctgtaagtcctgtttgagacccatagaa<br>gagatcaccctggactcaagtatggactacacgcccccagatgtatcccatgtgctgaagacatggaggaatggggaaggtt<br>cgtggggacaagagataaaacagatctatcctttagaagggaattggaagaatttagcacctgctgagcaatcctatcaagtc<br>ggcagatgtataggttttctatatggagacttggcgtatagaaaatctactcatgccgaggacagttctctatttcctctatctataca<br>aggtcgtattagaggtcgaggtttcttaaaagggttgctagacggattaatgagagcaagttgctgccaagtaatacaccggag<br>aagtctggctcatttgaagaggccggccaacgcagtgtacggaggtttgatttacttgattgataaattgagtgtatcacctccatt<br>cctttctcttactagatcaggacctattagagacgaattagaaacgattccccacaagatcccaacctcctatccgacaagcaa<br>ccgtgatatggggggtgattgtcagaaattacttcaaataccaatgccgtctaattgaaaagggaaaatacagatcacattattca<br>caattatggttattctcagatgtcttatccatagacttcattggaccattctctatttccaccaccctcttgcaaatcctatacaagccat<br>ttttatctgggaaagataagaatgagttgagagagctggcaaatctttcttcattgctaagatcaggagaggggtgggaagaca<br>tacatgtgaaattcttcaccaaggacatattattgtgtccagaggaaatcagacatgcttgcaagttcgggattgctaaggataat<br>aataaagacatgagctatccccccttggggaagggaatccagagggacaattacaacaatccctgtttattatacgaccacccc<br>ttacccaaagatgctagagatgcctccaagaatccaaaatcccctgctgtccggaatcaggttgggccaattaccaactggcg<br>ctcattataaaattcggagtatattacatggaatgggaatccattacagggacttcttgagttgtggagacggctccggagggat<br>gactgctgcattactacgagaaaatgtgcatagcagaggaatattcaatagtctgttagaattatcagggtcagtcatgcgagg<br>cgcctctcctgagccccccagtgccctagaaactttaggaggagataaatcgagatgtgtaaatggtgaaacatgttgggaat<br>atccatctgacttatgtgacccaaggacttgggactatttcctccgactcaaagcaggcttggggcttcaaattgatttaattgtaat<br>ggatatggaagttcgggattcttctactagcctgaaaattgagacgaatgttagaaattatgtgcaccggattttggatgagcaa<br>ggagttttaatctacaagacttatggaacatatatttgtgagagcgaaaagaatgcagtaacaatccttggtcccatgttcaaga<br>cggtcgacttagttcaaacagaatttagtagttctcaaacgtctgaagtatatatggtatgtaaaggtttgaagaaattaatcgatg<br>aacccaatcccgattggtcttccatcaatgaatcctggaaaaacctgtacgcattccagtcatcagaacaggaatttgccagag<br>caaagaaggttagtacatactttaccttgacaggtattccctcccaattcattcctgatccttttgtaaacattgagactatgctaca<br>aatattcggagtacccacgggtgtgtctcatgcggctgccttaaaatcatctgatagacctgcagatttattgaccattagcctttttt<br>atatggcgattatatcgtattataacatcaatcatatcagagtaggaccgatacctccgaaccccccatcagatggaattgcac<br>aaaatgtggggatcgctataactggtataagcttttggctgagtttgatggagaaagacattccactatatcaacagtgtttagca<br>gttatccagcaatcattcccgattaggtgggaggctgtttcagtaaaaggaggatacaagcagaagtggagtactagaggtga<br>tgggctcccaaaagatacccgaatttcagactccttggccccaatcgggaactggatcagatctctggaattggtccgaaacc<br>aagttcgtctaaatccattcaatgagatcttgttcaatcagctatgtcgtacagtggataatcatttgaaatggtcaaatttgcgaag<br>aaacacaggaatgattgaatggatcaatagacgaatttcaaaagaagaccggtctatactgatgttgaagagtgacctacac<br>gaggaaaactcttggagagattaaaaaatcatgaggagactccaaactttaagtatgaaaaaaactttgatccttaagaccct<br>cttgtggtttttatttttatctggttttgtggtcttcgt
